# Abstracts of papers and posters presented at the 2001 Pittsburgh Conference

**DOI:** 10.1155/S1463924601000190

**Published:** 2001


Journal of Automated Methods & Management in Chemistry, Vol. 23, No. 5 (September–October 2001) pp. 137–166

### Abstracts of papers and posters presented at the 2001 Pittsburgh Conference

The following 76 abstracts form Part B of two issues of *Journal of Automated Methods & Management in Chemistry* devoted to abstracts of papers and posters presented this year at the 52nd Pittsburgh Conference, held from 4 to 9 March 2001 in New Orleans, LA, USA. The papers and posters covered a range of topics and techniques, each of which provided valuable information to the conferees and exhibitors alike. Unfortunately, not all of the speakers provided abstracts, so I have been limited to those that were included in the book of Abstracts. This presents a worrying trend, which, added to the disappointing attendance figures, should give the organizing committees food for thought at least. Pittcon is my favourite show and has done a great job for analytical chemistry as a whole. Perhaps people are tiring of New Orleans as a venue, or is it becoming little more than a trade show? Hopefully, it will revive itself when the next show is held from 17 to 22 March 2002, also in New Orleans.

If you need further information about any of these abstracts please contact the authors. It is my intention to publish full papers corresponding to some of these abstracts in future issues of the journal.

Peter B. Stockwell
*Editor*

### Building sensory advantages in a food factory using the new αGEMINI electronic nose

*V. Schmitt, E. Chanie, H. Amine and J.-F. Chauvet, Alpha M. O. S. SA, 20 avenue Didier Daurat, F-31400 Toulouse, France*

Organoleptic characteristics of incoming materials and final products are essential to the food and packaging industry to assess product integrity and meet customer acceptance. The increasing number of controls by factory human panel have led to the development of a fast, accurate and high-quality electronic nose for comparison of pure and finished product control with 'gold references'.

The new algorithm and the principle of operating of the instrument to train the system on the gold reference will be presented. The performance of the system will be addressed in terms of accuracy and reproducibility and high throughput. Several applications will be presented to illustrate the capability of the electronic nose αGEMINI not only to assess product integrity, but also to follow process quality over the time for SPC/TQA in production facilities.

An example of the use of the αGEMINI system has been obtained from a 'coffee factory'. The system has been trained by analysing 10 different batches collected during 2 months of production. After this step, the quality variability could be represented by a line so-called 'warning limit' as shown in the figure. Then, several coffee batches obtained during the next 10 months have been analysed. The results have been projected on the SPC/SQC plot following the first training sample set results. The acceptability of the finished product is obtained when the unknown samples are located below the warning limit.

The αGEMINI's ability to run a sensory test in <5 min per sample and provide objective results allows companies to select better raw materials and to manufacture more consistently finished products.

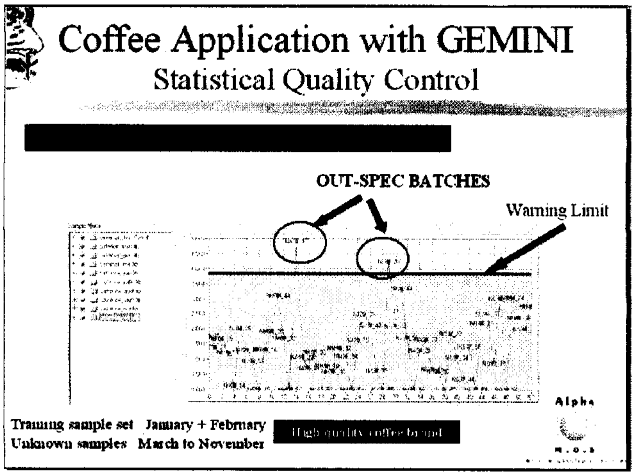

### Fish freshness assessment with an electronic nose directly at the fishing dock

*J. C. Mifsud and J.-Y. Bouteraon, Alpha M. O. S. SA, 20 avenue Didier Daurat, F-31400 Toulouse, France*

The fish industry is one of the largest food raw materials industry in the world with billions of dollars of transactions every years. Just in the USA alone, several billions of dollars of transaction involved mainly shrimps (35% of imports), salmon (17%), and catfish and crabs.

Several quality standards exist in the world to assess fish freshness such as the colour of the eyes and the curb of the belly. In Europe, fish quality is ranked in four major quality standards (A best quality to E, the worst). Quality is mainly linked to freshness and to the mechanical integrity of the fish. Seafood inspectors throughout the world would like to have instruments that would allow them to assess quickly the various quality of fish. The challenges of the task include the harsh environment and the high sample throughput required.

An instrument that associates an electronic nose equipped with 18 metal oxide sensors and a CCD camera has been used in several fishing harbours in Europe to recognize the various fish and their qualities. Excellent results and a good correlation over 1 month have been obtained. Results and sampling procedure will be discussed.

### Application of chemometrical methods in flavour analysis

*Detlef Ulrich and Edelgard Hoberg, Federal Centre for Breeding Research on Cultivated Plants, Institute for Quality Analysis, Neuer Weg 22/23, D-06484 Quedlinburg, Germany*

Most of the flavours appreciated today in fruit and vegetables were recognized many years ago and are often best represented in old varieties unsuited to large-scale commercial production. In addition, long-term plant breeding can cause a depletion of aroma patterns by genetic shift. Rapid and reliable analysis methods are required to integrate objective criteria for sensory qualities in plant breeding and quality assessment.

The topic of the presentation is the application of statistical techniques in combination with standard instruments, fast-GC and mass spectrometer, for electronic nose measurements in food research and industry. Examples of aroma analyses for strawberries, melons and boiled potatoes in dependence of their genetic background and/or the post-harvest treatments will be presented. The sensory quality of boiled potato extracts was checked by a combination of fast-GC with chemometric *data* processing. The statistical analysis of time sections of chromatograms with full chromatographic separation was used to differentiate between genotypes with varying sensory qualities. In contrast to potatoes, the aroma patterns of various strawberry and melon genotypes were investigated by using mass spectrometric sensor measurements as a reliable method. Fruit homogenate volatiles were sampled by headspace SPME with a PDMS/DVB fibre and injected in a GC/MS system equipped with a short, unpolar column. No chromatographic separation was necessary to use the mass spectrometric detector as sensor. A chemometric software was applied for data processing using the mass fragments from $m/z$ 27 to 300 amu as 'sensors'.

Both methods characterized here are capable of differentiating between different flavour patterns in fresh fruits or boiled vegetables. Because of their simplicity and rapidness, the methods are applicable as screening techniques in plant breeding as well as quality assessment.

### Combinatorial and high throughput methods for materials R&D: an advanced technology program technology cluster

*John D. Hewes, Advanced Technology Program, National Institute of Standards and Technology, 100 Bureau Drive, Gaithersburg, MD 20899-4730, USA*

The National Institute of Standards and Technology (NIST) Advanced Technology Program (ATP) provides funding on a cost-share basis to industry for high-risk/high-payoff research on emerging and enabling technologies. The ATP concentrates on technologies that offer significant, broad-based benefits to the nation's economy, but that are unlikely to be developed in a timely fashion without ATP support. The ATP is currently funding research projects in high-throughput discovery of catalysts and polymer coatings, with FY1999 and 2000 project budgets of $44 million (ATP share $26 million and industry cost-share) $24 million over 5 years.

The NIST Measurement and Standards Laboratory (MSL) also has an exciting opportunity in high-throughput experimentation: to develop measurement science to support new parallel methodologies and measurement tools tailored to specific industrial needs; to validate new and existing measurement methods and models using parallel or high-throughput approaches; to demonstrate application of HT methods to new materials and R&D problems; and to develop new standards addressing systems integration issues. The ATP supplements the NIST MSL through its intramural research program and is funding research in scanning microwave microscopy of BST thin layer dielectrics, genetic programming for data mining and visualisation, and two-dimensional mid- and far-FTIR imaging.

### Minimizing validation effort in analytical data systems using automated regression test tools

*Wolfgang Winter, Agilent Technologies GmbH, Lifescience Business Unit, Hewlett-Packard Str. 8, D-76337 Waldbronn, Germany*

Systems compliant with Part 21 CFR Part 11 must be validated. However, without adequate qualification procedures and tools, the qualification and requalification of systems can become a labour-intensive task. In many cases, system validation becomes more expensive than the initial purchasing price.

This paper discusses how automated regression test tools become invaluable during systems development and test, and how the same approach can be applied to perform other parts of the system validation effectively and consistently. This is particularly true in the area of revalidation of a system, e.g. after installing a new software revision update.

By applying state-of-the-art software design methodology, the difficulties with changing software and hardware platforms become manageable. Proper encapsulation of the algorithmic portions of a system from the user interface and other operating system-dependent layers of a networked data system minimizes the dependencies on a particular system environment. This (object-oriented) method of structuring a software system enables portable algorithms that can be migrated across generations of systems or even in new ones specifically designed for a different operating system.

This encapsulation approach also allows test-engineering teams employed by the manufacturers to develop powerful computer-based, automated regression test suites. Even in the early stages of a software product's lifecycle, these test suites allow the vendor to perform extensive black- or white-box tests on a particular module.

Known input can be fed into the interfaces of the module and the resulting output can be compared against predefined test specification results. The regression test software can automatically determine whether the result is within the defined acceptance limits and flag deviations if they occur. The same technique can be applied for system qualification at installation and for continuous performance verification in the analytical laboratory. This works not only for software qualification, but also for qualification of analytical instrumentation. Since the regression tests are computer-based, the delivery of the qualification is consistent and fast compared with the manual execution of a paper-based test protocol.

Conclusion: state-of-the art networked data systems should use automated regression testing tools. This technology not only helps to increase system reliability and quality during the development phase, but also enables consistent and fast execution of system qualification (IQ, OQ/PV) in the analytical laboratory.

### Analytical method validation: an automated software approach

*Patricia A. Fowler and Michael Swartz, Waters Corp., 34 Maple Street, Department TG, Milford, MA 01757, USA*

Method validation is a tedious process performed to determine if an analytical method meets the requirements for the intended purpose. In the regulated laboratory, method validation may take many days to perform the analytical tests necessary to evaluate linearity, accuracy, precision, limit of detection, limit of quantitation, specificity and robustness. These parameters must be evaluated to determine if a particular method truly exhibits the performance characteristics that are expected by regulatory agencies such as the FDA. The data reduction and statistical analysis performed on these analytical tests can be the most time consuming and tedious part of the process. There is also a greater possibility of introducing error, especially when calculations are performed manually. With the use of automated software to perform these calculations, an analytical method can be validated much faster and easier, with less chance for error.

The analytical method in this presentation was validated using automated software to determine linearity, accuracy, precision, LOD, LOQ, specificity and robustness. Chromatographic results are directly accessed in a relational database, bypassing manual intervention. Statistical calculations were performed automatically with a report generated showing the results of the analyses from the Student, Cochran, Dixon, and Fisher tests. Graphs representative of the statistical analysis obtained while validating this method will be shown. The data reduction and statistical calculations necessary to validate the method, complete with the necessary documentation and report generation was completed in significantly less time. This presentation will highlight the advantage in time savings and error reduction, as well as improvements in efficiency and the decision-making process for method validation.

### Practical application of selenium speciation in process discharge streams

*Ruth E. Wolf[1], Dirk Wallschliger[2] and Nicolas S. Bloom[2], [1]PerkinElmer Instruments, 761 Main Avenue, MS-219, Norwalk, CT, [2]Frontier Geosciences, Inc., 414 Pontius Avenue North, Seattle, WA, 98109, USA*

Owing to the different uptake and bioaccumulation rates and ecotoxicity of various selenium compounds, the identification and measurement of each compound is becoming increasingly important. Many industries face challenges in determining the levels of selenium species present and the best method for remediation of toxic species. The industries affected are widespread and include zinc, lead and gold mines, phosphate producers, power plants, coal mines, refineries, glass manufacturers,

*Method validation of triamterene: summary report.*

Analyst: system — Date: 21-Sep-2000 9/21/00 15:42

*The following answers are asserted with a confidence level of 0.95 or a risk level Alpha = 0.05*

**Linearity and range**

| | | | | | | | | |
|---|---|---|---|---|---|---|---|---|
| #Points= | 15 | 5 | Groups | (concentrations)* | | 3 | Replicates | (days) |
| Range(%): | 70% | 130% | Pharm. /Bio. /Sample | | | Active form / Standard | | |
| 100% = | 50.0 mg | | Slope: | 45097.6 | | Slope: | 44454.35971 | |
| Weighting factor: 0 | None | | Intercept: | −40296.6 | | Intercept: | −17092.90221 | |
| | | | Cor. Coeff. | 0.99967 | | Cor. Coeff. | 0.99985 | |
| Is the Intercept through 0? | | | No T test | 2.16 | 2.33 | Yes T test | 2.16 | 1.47 |
| Are the variances homogeneous? | | | Yes C test | 0.68 | 0.5 | Yes C test | 0.68 | 0.35 |
| Is the slope significantly <> 0? | | | Yes F test | 4.67 | 19652.88 | Yes F test | 4.67 | 42318 |
| Is the system response linear? | | | Yes F test | 3.71 | 2.56 | No F test | 3.71 | 7.42 |
| Are the two Intercepts significantly different? | | | | | No | T test | 2.06 | 1.11 |
| Are the two Slopes significantly different? | | | | | No | T test | 2.06 | 1.66 |

The Calibration curve of the 100% Standard should be considered as the reference.

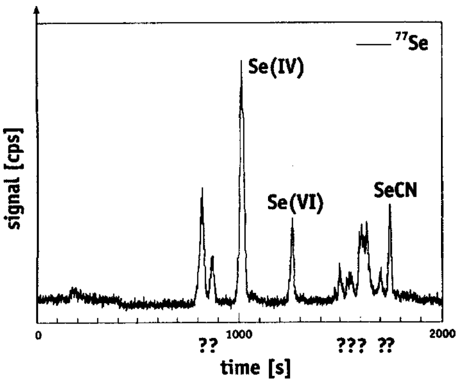

*Separation and determination of known and unknown Se species in a mixture of industrial waste waters by IC-ICP-MS.*

and agricultural irrigators. The toxicity and clean-up methods vary, depending on the specific selenium species present and the environment into which they are released. For example, Se(VI) is fairly persistent in water. On the other hand, Se(IV) has a higher affinity for particulate matter and is easily reduced by bacteria, making it much more prone to removal from the water by moving into the sediment.

Past approaches to speciate selenium have resulted in limited success when transferred to real sample matrices. Operationally defined methods fail because of the interferences that can occur in complex matrices and the presence of unanticipated species (such as selenocyanate or organoselenium species). The use of ion chromatography (IC)-ICP-MS and IC-hydride generation (HG)-ICP-MS techniques for performing selenium speciation in a variety of waste process streams has been evaluated and will be discussed. Besides the commonly encountered species selenite and selenate, various currently unidentified Se species are found in many industrial waste water streams (see the figure). Data showing the effect of common sample characteristics, such as salinity, on the method performance will be presented. Recommendations regarding methodologies developed for particular process streams will be presented and discussed.

### Design of continuous monitoring of analytes by membrane extraction with sorbent interface

*Ionel Ciucanu and Janusz Pawliszyn, Department of Chemistry, University of Waterloo, Waterloo, ON, N2L3G1 Canada*

The importance of continuous, fast and portable instrumentation is gaining more attention in the recent years. The most important feature of any continuous instrumentation is the sample introduction device, which must make a automatic and reproducible injection. Membrane extraction with sorbent interface (MESI) is such a technique that has no moving piece, can inject a large volume of sample without band broadening, and is solvent free, simple, fast and inexpensive.

In this communication, the development of a new design of MESI system composed of membrane module, microtrap and heated line transfer will be presented. Each component of the system will be described. The analytes were extracted from sample matrix with a very sensitive

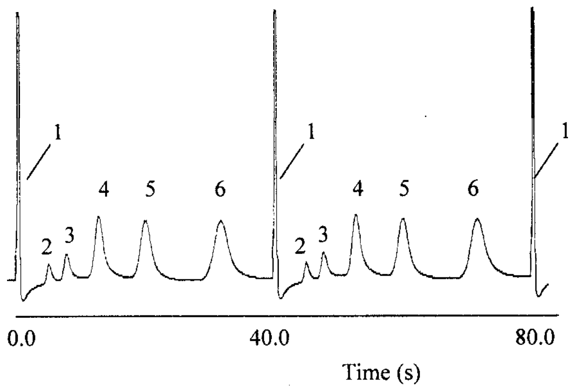

*Gas chromatograms for the continuous monitoring. 1, Air; 2, benzene; 3, trichloroethylene; 4, toluene; 5, tetrachloroetylene; 6, ethylbenzene.*

membrane module. The extracted analytes were concentrated in a very small amount of sorbent of the microtrap and were periodically desorbed by applying an electric/thermal pulse. The desorptions were performed at fixed intervals, corresponding with an injection in a capillary column. A heated silicosteel capillary column was used as a transfer line avoiding adsorption and condensation of analytes, performing a first separation of the analytes, improving the resolution and making the process faster. Using a microGC and a 12 V DC power supply made the system portable.

The MESI system designed was suitable for continuous analysis of solid, liquid and gaseous sample in laboratory, field and in process control applications. Continuous monitoring of aromatic hydrocarbons, chlorinated compounds and terpenoids from different sources with this MESI system will be presented.

### Field-based material characterisation with portable FTIR spectroscopy

*John P. Coatis, S-Coates Consulting, Newtown, CT 06470, USA*

For years, as analytical chemists, we have accepted the fact that we have to perform detailed analyses for sample characterisation and identification in a laboratory. This is particularly, the case with techniques such as infrared spectroscopy, which today typically requires a standard FTIR spectrometer equipped with one or more sample-handling devices (or accessories). At times there are problems associated with taking samples back to the laboratory, such as the potential for loss and/or misdirection of a sample, the opportunity for mix-ups between samples, and even the risk of sample degradation and/or adulteration. Other initiations can be restrictions imposed on the transportation of materials. Good examples include criminal investigations, where the optimum forensic solution would be the ability to perform the analysis at the crime scene, and on-site testing of waste, especially for recycling and/or hazardous material disposal.

This paper will present a new concept of on-site material characterisation, where a universal sampling aid is integrated with a compact transportable FTtR system, allowing rapid identification of solid or liquid materials. Practical examples for both forensic sample identification and waste material characterisation will be presented.

### Automated spectral interpretation of surface-enhanced Raman spectra collected from landmine vapor signatures

*James A. Janni, James M. Sylvia, J. D. Klein and Kevin M. Spencer, EIC Laboratories, Inc., 111 Downey Street, Norwood, MA 02062, USA*

The interpretation of surface-enhanced Raman spectra (SERS) has often depended on visual analysis by a SERS specialist. Variable background emission, frequency-shifted Raman bands and bands from other substances complicate SERS spectral evaluation. Despite these hurdles, SERS has emerged as a highly sensitive and selective method for detection of nitro-explosives.

The utility of SERS for explosive detection has been enhanced with the development of spectral analysis tools based on principal component analysis (PCA). The automated PCA outperformed the visual inspection protocol in substantially less evaluation time. A 96% successful identification rate with 14% false-positive rate was achieved in <10 s of analysis time for 54 landmine simulants. Visual inspection took hours longer for a lower positive identification rate and a higher false-positive rate. Vapor concentrations of 2,4-DNT, the analyte for these tests of SERS landmine detection, ranged from 50 to 5 ppb and were sensed with electrochemically roughened gold coupons.

Based on estimates of the total analyte available and the collection efficiency of the SERS probe, SERS spectra were collected from <1 pg 2,4-DNT. PCA also provided a qualitative measure of substrate quality with obvious implications for improved substrate synthesis. In a separate test of SERS sensitivity to landmine vapors from contaminated soils, the principal components separated into positive and negative indications for landmine and for poor substrate sensitivity. This second test of landmine sensitivity used samples contaminated with highly aromatic diesel fuel. Aromatic compounds are highly SERS active, yet the PCA approach to sample characterisation distinguished between the diesel interferents and the 2,4-DNT signature spectrum.

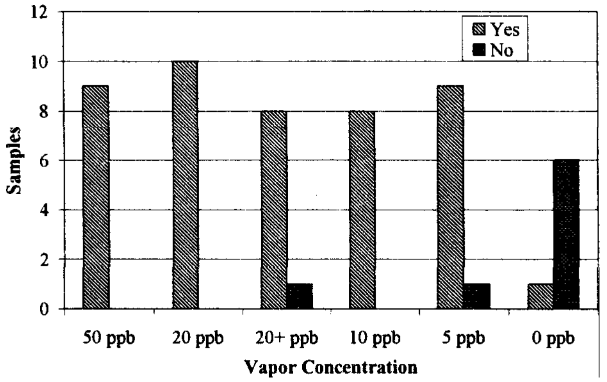

*Characterisation of the 54 landmine simulants presented show a significant demonstration of the sensitivity and reproducibility of SERS.*

### Advances in spectroscopy: spectral database searching on the Internet

*James Duckworth[1], Joni Hansen Miller[2] and Chris Draves[2], [1]Galactic Industries Corp., 395 Main Street, Salem, NH 03079-2464, [2]Thermo Nicolet Corp., 5225 Verona Road, Madison, WI 53711-4495, USA*

Since 1995, there has been explosive growth in the use of the Internet by the general public. The penetration of the Internet into society is also fundamentally changing the face of scientific, chemical and spectroscopic research. For spectroscopists, this has meant better access to publications, information and specifications on commercial instrumentation. Within the last year, on-line services have made the Internet not only more than just an information tool, but also a valuable resource for solving problems that arise in the laboratory. For example, FTIRsearch.com was introduced as a service that allows spectroscopists to compare their spectra with commercial spectral databases over the Internet.

Until about 15 years ago, spectroscopists spent hours searching through books of spectra to attempt to match visually the spectra they measured from their samples. With computerisation of spectrometers came the ability to perform rapid and reliable spectral searching against large digital databases of spectra. At the time, this was a revolutionary development as it dramatically reduced the time it took spectroscopists to identify compounds.

However, it was still time consuming to measure all the necessary compounds to build up the digital libraries. Owing to this, a new commercial industry evolved to create database search software and spectral libraries. There is a substantial amount of work involved in developing high commercial-quality collections of spectra. While the information value to spectroscopists is very high, not everyone could afford them. This was especially true of smaller laboratories, researchers involved in short-term projects, educational institutions where the need to search spectral databases is infrequent.

Now in the age of the Internet, where it is possible to access powerful computers through a simple Web browser, comes the next revolution in searching spectral database; *on-line access*. FTIRsearch.com takes advantage of the unparalleled connectivity of the Internet to offer every chemist and spectroscopist access to large, commercial-quality FTIR and Raman spectral databases at affordable prices.

The Internet is the ideal way to make use of spectral databases. Since it is always ‘on’, there is never a need to locate a computer that has software or libraries. As more and more people have access to the Internet at work, the ability to connect multiple users around the world to a single, powerful computer makes it the obvious choice for delivering a comprehensive spectral database search product.

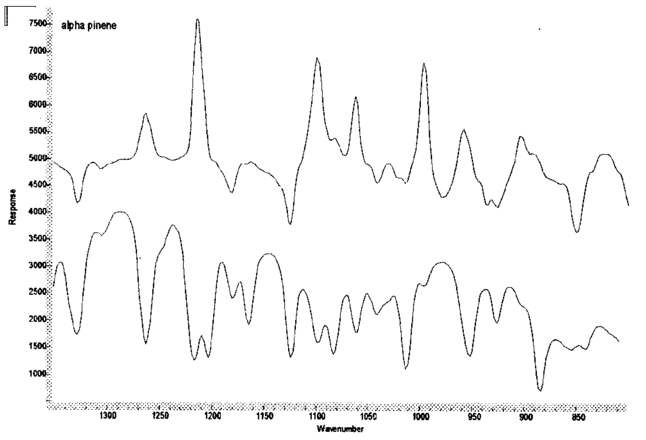

*Vibrational circular dichroism spectrum (top) and single-beam spectrum (bottom) of α-pinene collected with DSP software without the use of a lock-in amplifier for signal demodulation.*

### Digital signal processing techniques applied to multiple modulation FTIR measurements

*David L. Drapcho, Raul Curbelo, Richard A. Crocombe and David Johnston, Bio-Rad Laboratories, Digilab Division, 237 Putnam Avenue, Cambridge, MA 02139, USA*

In previous papers we demonstrated the use of digital signal processing (DSP) software algorithms for demodulating the phase-modulation component in step-scan FTIR photo-acoustic measurements [1], and the simultaneous demodulation of both the phase modulation and sample modulation components in dynamic polymer stretching measurements [2]. These software techniques eliminate the requirement for one or more lock-in amplifiers for demodulation of the component signals, thereby eliminating the extra cost burden and experimental complexity of these devices. In addition, DSP software techniques have been shown to provide increased sensitivity by allowing the full use of the dynamic range of the FTIR spectrometer. This paper will discuss the continued extension of the DSP methods to triple modulation measurements, specifically those measurements that employ a photo-elastic modulator in combination with phase modulation of the spectrometer (US Patent 6,025,913). These techniques will be applied to dynamic infrared linear dichroism, vibrational linear dichroism (VLD) and vibrational circular dichroism (VCD) measurements. Examples of these applications will be discussed, contrasting the benefits of this approach to demodulation with a series of lock-in amplifiers.

1. Drapcho, D. L., Curbelo, R., Jiang, E. Y., Crocombe, R. A., and McCarthy, W. J., 1997, *Applied Spectroscopy*, **51**, 453.
2. Drapcho, D. L., Curbelo, R., Crocombe, R. A., Zhang, L., and Johnston, D., 1997, Pittsburgh Conference, Atlanta, GA, paper 107.

### Field application of the particulate matter characterisation and monitoring system for the real-time evaluation of emission particles from combustion sources

*Paul Nam[1], Hongyu Shen[2], Shubhen Kapila[1,2], Philip Whitefield[1,4], Don Hagen[3,4] and Steve Chelli[5], [1]Center for Environmental Science & Technology, [2]Department of Chemistry, [3]Department of Physics and [4]Cloud and Aerosols Science Lab, University of Missouri-Rolla, MO 65409, [5]Deposition Research Laboratory, Inc., St Charles, MO 63301, USA*

A particulate matter characterisation and monitoring system (PMCMS) consisting of a tandem arrangement of a particle size analyser and a mass spectrometer was assembled in a mobile platform for field testing of combustion sources such as aircraft jet engines. The on-line particle analysis system was used for on-line real-time particle size and chemical composition determinations of particles emitted by prototype engines and combustors at US Air Force and NASA installations.

Characteristics of emitted particles were monitored under varied operating conditions. The characterisation of particles emitted from an angular sector combustor rig at the NASA Glenn Research Center involved size distribution analysis and detection of metals borne by the submicron particle. Of 41 elements monitored, most prevalent were Al, Si, Ca and Fe. These elements were detected in the emission particles during all operational conditions. They are most likely originated from the alumina refractory housing of the combustor. In contrast, no zirconium was detected in the emission particles, although the combustor also contains refractory made mainly with zirconium oxide.

The real-time particle size distribution and elemental analyses of combustion particles emitted by a T-63 Gas Turbine Engine were performed at the Wright-Patterson Air Force Base ARL Fuels Branch. A representative broad size distribution and elemental composition of the particles emitted by the engine is shown in the figure. The PMCMS detected several metals in the particles, and the most abundant metals were chromium and vanadium.

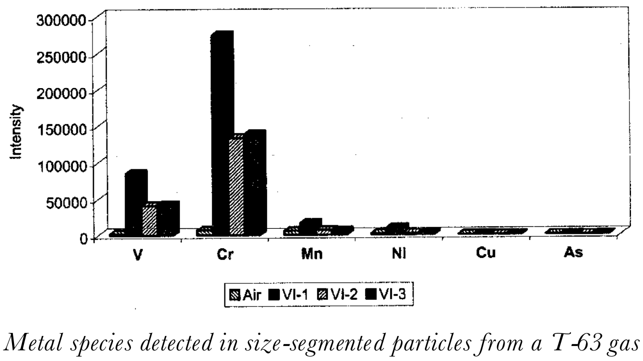

*Metal species detected in size-segmented particles from a T-63 gas turbine engine operated with a fuel additive (air: filtered air, VI-1: particle diameter, 0.25–1.0 μm, VI-2: particle diameter 1.0–2.5 μm, VI-3: particle diameter 2.5–10 μm).*

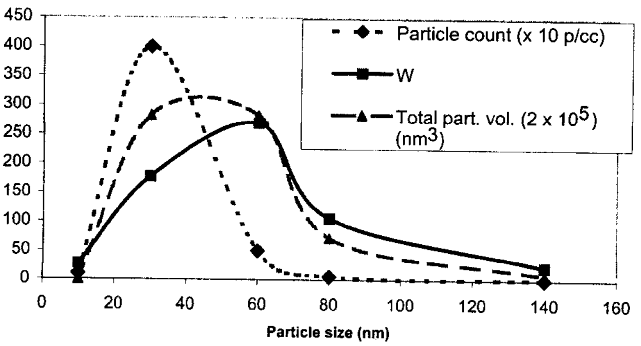

*Real-time characterisation of submicron particles produced from a resistively heated tungsten wire.*

### On-line characterisation of aerosols generated from electrically heated metal wires

*Hongyu Shen[1], Paul Nam[2], Shubhen Kapila[1,2], Philip Whitefield[1,4] and Don Hagen[3,4], [1]Department of Chemistry, [2]Center for Environmental Science & Technology, [3]Department of Physics and [4]Cloud and Aerosols Science Lab, University of Missouri-Rolla, MO 65409, USA*

Fine (submicron) particles generated by Joule heating of metal wires in gas streams have been used as test aerosols in diverse research ranging from development and validation of aerosol monitoring instrumentation, particle deposition and health effect monitoring. Although shape and size characteristics of the submicron particles have been studied extensively, accurate information on the chemical constituents of the particles has been limited, primarily due to the difficulties in sampling and analysis of fine particles. An on-line particle characterisation system consisting of differential mobility analyser (DMA) and an inductively coupled plasma-mass spectrometer (ICP-MS) was developed and used for characterisation of submicron particles emitted by electrically heated metal wires. The system permitted an on-line, real-time particle size and chemical composition determination of aerosols produced from electrically heated wires. When nichrom wire consisting of 60% Ni, 20% Cr and 20% Fe was resistively heated, the submicron particles found in gas streams were largely composed of pyrolysis products of residual organics in the gas streams. As a result, except under highly sterile conditions, little correlation between metal concentration and number distribution of aerosols could be observed. However, a good correlation between total particle volume and W concentration was readily obtained with a resistively heated tungsten wire (see the figure).

### Strategic approach to creating robust chemometric methods

*R. A. Hoult, D. P. Lidiard and R. A. Spragg, PerkinElmer Ltd, Post Office Lane, Beaconsfield HP9 1QA, UK*

Ideally, chemometric methods would be freely transferable between instruments and insensitive to any changes over the lifetime of an instrument. This is quite readily achieved for some applications, but problems can arise when methods rely on very subtle spectral differences. We describe a three-component strategy that addresses this issue within a system for identity and conformance testing by FTIR and FTNIR.

The first step is to control the spectrometer hardware as closely as possible. However, component and manufacturing tolerances limit what can be achieved in practice. A significant further improvement can be obtained by using software to standardise the instrument response function, that is the wavelength calibration and the lineshape. For this we have adopted the Absolute Virtual Instrument approach in which a mathematically defined 'ideal' instrument is used as the reference standard. It involves measuring the spectrum of a low molecular weight gas and deriving a transfer function that converts this to what would be obtained from the ideal spectrometer. This transfer function is then applied to all spectra as they are measured. By using a standard sample built into the spectrometer, it is possible to characterise the instrument with any sampling system in place. The calibration can be updated at any time to handle any effects of aging or replacement of components.

The third component aims to address variations that arise at the sampling interface, for example NIR reflectance or ATR. Our approach is to identify and quantify these variations by analysing data collected from many systems during manufacture. Principal component analysis has proved very effective for this. Major variations are modeled and can then be introduced automatically into chemometric methods. This eliminates or greatly reduces the sensitivity of methods to these variations.

### Automated near-infrared diffuse reflectance and transflectance analysis of liquids: from laboratory to process

*Arnold J. Eilert, Robert Gajewski, Mark Weseman and Doug McCutcheon, Bran & Luebbe, Inc., 1025 Busch Parkway, Buffalo Grove, IL 60089, USA*

Near-infrared (NIR) analysis of liquids can be relatively straightforward when sample temperature is constant, scattering losses are low and transmission-mode path lengths can be optimised. However, with highly scattering liquids, simple transmission mode measurements may not be possible. In addition, the temperature of the product may vary considerably, especially with process applications. NIR spectrometers and sample-handling systems have been developed to deal with these complications. In some cases, diffuse reflectance can simply be employed. This may be the method of choice if the material is highly reflective with sufficiently consistent scattering efficiency.

However, in many instances, additional control of sample presentation (including sample thickness and temperature) is necessary. Automated transflection-mode flow-through measurement systems have been developed for such applications. These systems allow cell thickness to be optimised for a particular application and provide pre-analysis sample temperature conditioning. When sufficiently rugged and reliable technologies are implemented in the laboratory, methods developed using the laboratory-based measurement systems can be readily transferred to process measurement systems for on-line monitoring. Various applications in the food and beverage industry will be presented.

### Applications of automated mass spectrometry in gemonics

*Charles R. Cantor, Sequenom, Inc., San Diego, CA, USA and Sequenom GmbH, Hamburg, Germany*

Sequenom has developed the technology to scan an array of samples in a chip format by matrix-assisted laser desorption ionisation (MALDI) time of flight (TOF) Mass spectrometry (MS). Current instrumentation uses samples that are 200 μm². About 6 fmol DNA in 6 nl is required; proteins. Other analytes require far less material. Mass spectra can be acquired automatically at up to 1.2 s per sample. Available apparatus can process 10 384 sample chips simultaneously using chips profiled with matrix DNA analysis by Sequenom's technology covers a gamut of applications from sequencing and allele detection to mutation finding and gene expression analysis. Allele determination is the most well-developed application and the one likely to enable the implementation of high-throughput genomics. MS analysis of alleles offers several advantages over more conventional methods. The assays are so robust that assay development can be automated. Thus, the large number of single nucleotide polymorphisms (SNP) required for a genomic study is not daunting. Assay development, SNP validation and the determination of allele frequencies in population pools can be carried out in a single experiment. If well-designed population pools are created, one can potentially drop the cost and speed the rate of associating alleles with diseases or other phenotypes by two to three orders of magnitude.

The much high resolution of MS allows a number of different polymorphic loci to be processed in a single spectrum. Such a procedure is called multiplexing. The high resolution also allows unequivocal detection of heterozygotes and in some cases even allows the phase of compound heterozygotes to be called. The overwhelming advantage of MS-based genotyping is its accuracy. Errors are never made in known alleles. Sometimes, MS actually reveals the presence of alleles that were not previously known.

Automated MS can also be used for mutation discovery or resequencing. This is done by fragmenting a sample enzymatically and detecting mutations as altered fragment weights compared with what was predicted from the known sequence. Fragmentation analysis is still in its infancy but it has the potential for reaching very high throughputs and should be useful both in DNA diagnostics and SNP discovery.

### Advances in automated structure verification using infrared spectroscopy

*Antony J. Williams[1] and Andrey Bogomolov[2], [1]Advanced Chemistry Development, 90 Adelaide Street West, Suite 702, Toronto, Ontario, Canada M5H 3V9, [2]Advanced Chemistry Development, Russian Office, 6 Bakuleva Street, Moscow 117513, Russia*

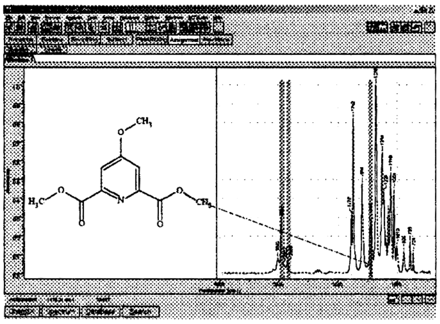

Infrared spectroscopy is no longer the principal tool of choice for structure elucidation and confirmation as in many cases it has been replaced by a combination of both mass spectrometry and nuclear magnetic resonance spectroscopy. While infrared spectroscopy has fallen out of favor, it still provides valuable information about functional group identification, which is often difficult to identify through other methods. Infrared spectroscopy does have the distinct advantage of being a simple experimental technique and the spectral features can be interpreted and compared with tables of standard vibrations to aid in the identification of specific functional groups. With the increasing pressure to *perform* sample analyses and confirm molecular structures in a short time, the ability to verify consistency between an infrared spectrum and a suggested structure in an automated fashion is motivating. Here we present our results in developing an integrated processing and databasing software suite for handling UV-Vis and infrared data with molecular structure integration. Hypothetical chemical structures and their consistency with their associated infrared spectra can be performed via an automated analysis of spectral features and functional group analysis. We believe this approach will be invaluable in the ongoing efforts to reduce sample analysis times.

### Automated detection of chemical vapors by multispectral infrared remote sensing imaging

*Mukire J. Wabomba and Gary W. Small, Center for Intelligent Chemical Instrumentation, Department of Chemistry and Biochemistry, Ohio University, Athens, OH 45701, USA*

There has been a growing desire to employ infrared (IR) remote-imaging technique in the detection of chemical vapor species. This desire calls for the exploration and development of automated detection techniques that result in quick and precise qualitative identification of individual analytes against a variety of infrared backgrounds and interferences. In this approach, multispectral infrared remote imaging, which has the ability simultaneously to collect both spatial and spectral measurements of a distant scene, is used.

The goal of this research is to develop an automated detection strategy to be used for identification of chemical vapor species in multispectral IR remote-imaging data in the atmosphere. The success of this work will be helpful in such applications as monitoring chemical weapons as well as environmental pollution from an airborne distant platform. This work utilises data from

RS-800 multispectral infrared line scanner (Raytheon TI Systems, McKinney, TX, USA) to image and detect chemical vapor species from an airborne platform. Signal processing, image processing and pattern recognition methods are used in the development of an automated detection system. In this presentation, ammonia, ethanol, methanol and sulfur dioxide are the analytes of interest. These chemical species are released from a hot stack and the plume emanating from the plume generator is imaged with the line scanner system.

### Automated, ultratrace, remotely controlled analysis of VOCs in ambient air

*James G. Moncur and John T. Whitechurch, Trace Analytical, 3517A Edison Way, Menlo Park, CA 94025, USA*

Instrumentation for automated analysis of volatile organic compounds (VOCs) in air needs to comply with government mandated goals and for clean-room monitoring. Analysis of air and other gases are achieved using built-in adsorption tubes and GC/FID. Concentrated air or other samples are desorbed into a capillary column system. One analyser is designed to detect C2 to C6 VOCs using an alumina-phase capillary column while another analyser detects C6 to C10 VOCs using a DB-624 phase capillary column.

PC-based software solutions for on-site control of the gas chromatograph and data collection allows instrument access via modem or other communication protocol. Raw peak information, chromatograms, substance tables, retention times and trend data are stored locally on the PC hard disk. Operating parameters and data can be reviewed and adjusted on site or remotely to improve quality of data and further reduce the cost of operation.

Automated, continuous analysis of ambient air avoids the high cost of canister-sampling methods and allows determination of air contaminants without delay. Ppb and ppt level detection of VOCs is permitted by adjusting the sample volume during trapping. Examples of applications will be discussed.

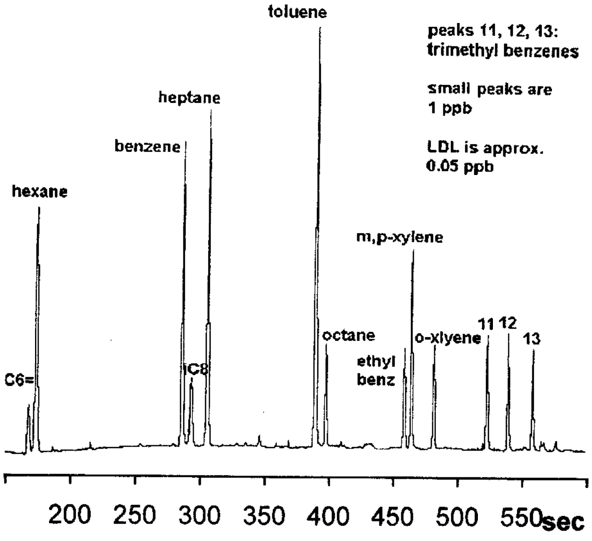

*Gas chromatogram of C6 to C9 VOCs in air at 1–5 ppb.*

### Fourier transform infrared spectroscopy in continuous emission monitoring: an update

*Vlastimil Karfik[1], Allan Rilling[2] and Bill Worthington[3], [1]Analytical Division of ABB Automation, Frankfurt, Germany, [2]Analytical Division of ABB Automation, Quebec, Canada, [3]Analytical Division of ABB Automation, Lewisburg, WV, USA*

FTIR spectroscopy was presented previously at Pittcon 1997 as a mature analytical method for industrial-level emission monitoring systems. In this update, the experience gained from 8 years of monitoring stack emissions will be presented as well as the operating principle and construction of the FTIR-based continuous emissions monitoring system (FTIR-CEMS). FTIR spectrometers designed for industrial environments have proven to fulfill the needs of waste and chemical incinerators by providing measurements of up to 10 chemical compounds simultaneously while the number is extendable up to 30 measurement components measured simultaneously. Performance, reliability and sensitivity are key parameters to the success of the FTIR-based CEMS.

The FTIR spectrometer with heated sample cell allows measurement of stack gas 'as is', thus avoiding any distortion of analytical results for water-soluble compounds like HCI, HF and $NH_3$. Statistics will be presented for the measurement of extremely low ppm levels of pollutants like HCI, $SO_2$, CO, NO, $NH_3$, HF, even in the presence of large amounts of water vapor.

The ruggedness, selectivity, long-term stability and simple design combine to fulfill regulations of the German Federal Ministry of Environment and Reactor Security, as well as other similar European Community regulations. Worldwide regulations continue to converge with the primary purpose of monitoring pollutants from incineration processes at decreasing levels with increased demand for reliability and up-time.

Users of the FTIR-based CEMAS emphasise criteria such as availability, accuracy and calibration stability. Calibration stability, for example, demonstrates the high-level stability for the CEMAS along with the advantage of limiting expenses due to consumption of calibration gas and cost of maintenance.

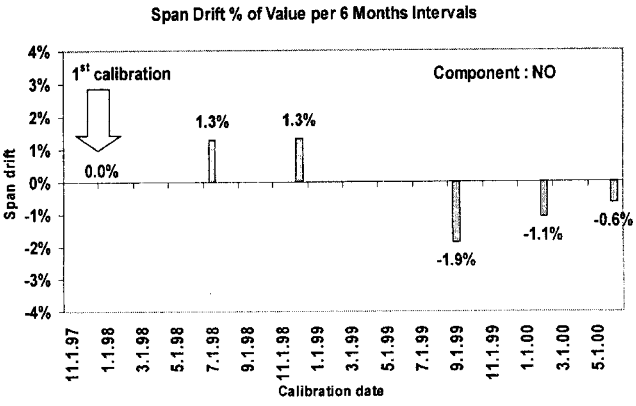

*Example for the calibration stability of the Advance Cemas-FTIR.*

The results obtained from the operation of more than 240 FTIR continuous emissions monitoring systems throughout the world will be discussed.

### Robust screening of dioxins and furans by ion-trap GC-MS/MS

*John D. Ragsdale and T. Michael Harvey, Thermo Finnigan, 2215 Grand Avenue Parkway, Austin, TX 78728, USA*

The determination of the presence of chlorinated dioxins and furans in the environment and foodstuffs has always been a great concern. Since there are no known safe levels of exposure to these substances, the only way to minimise risk is to minimise exposure. Therefore, only the most sensitive and specific instruments are capable of achieving the detection limits required for regulatory confirmation. These detection limits can be achieved by using high-resolution magnetic sector instruments capable of detecting the dioxins and furans by their exact mass. Not all samples require this level of detection.

Ion-trap mass spectrometers have long been known for their sensitivity and relative low cost compared with other types of mass spectrometers. Applying ion-trap MS/MS technology to the determination of dioxins and furans leads to a viable and robust screening technique that can be used for screening, quantitation and confirmation down to the low ppt range. This is achieved using MS/MS for specificity and using a split injection for increased chromatographic robustness.

The figure depicts the analysis of extracted palm oil that has been spiked with 2,3,7,8-tetrachloro-dibenzo-dioxin and furan at 1 pg $\mu l^{-1}$. The precursor and product ion masses for the dioxin and furan are $320 + 322$, $257 + 259$ and $304 + 306$, $241 + 243$, respectively.

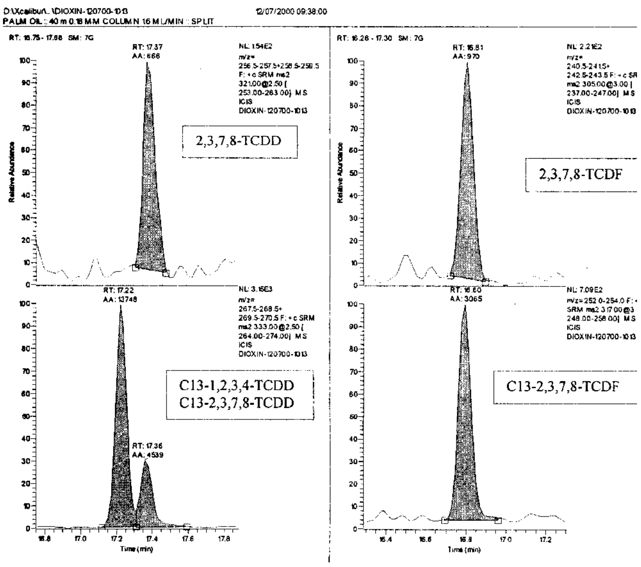

*Analysis of extracted palm oil spiked with 2,3,7,8-tetrachloro-dibenzo-dioxin and furan at 1 pg $\mu l^{-1}$.*

### Semi-automated sample deposition system

*Richard A. Todebush and James A. De Haseth, Department of Chemistry, University of Georgia, Athens, GA 30602, USA*

The detection of minute samples is a concern in many areas of research and analysis, including biological, environmental and forensic science. The use of manual solution direct deposition, combined with surface evaporation, is a very useful and convenient method for the transfer of an analyte to a spectroscopic sampling window. This transfer is most commonly performed with the use of a small pipette and manual placement of the analyte within a specific area. In the case of Fourier transform infrared (FT-IR) spectrometry, a microscope is used to focus the radiation onto the sample deposit. This method, however, can present a problem with the thickness variability of the deposit. The tendency is for the sample to dry thicker around the edges than in the middle of the deposit, which can make the focusing of the microscope beam onto a 'usable' area of the deposit somewhat of a difficult task, especially if the total deposit diameter approaches the spatial resolution of the microscope.

One method used in our laboratory to alleviate the sample thickness concern is to use a vacuum apparatus to aid in solvent evaporation during deposition. A glass pipette is attached to a vacuum line and placed ~3 mm above the center of the deposition area. This allows air to be drawn up and over the sample deposit, thus concentrating the sample into a smaller area. The result of this method is an area of more uniform sample thickness on which to perform FT-IR transmission spectroscopy. An alternative method to transmission spectrometry is the use of a single-bounce attenuated total reflection (ATR) element attached to an FT-IR spectrometer. The Harrick Split-Pea TM improves sensitivity by focusing the infrared beam onto a very small area, ~250 μm in diameter. With the beam focusing and sample 'containment' problems resolved a new concern arises. With such a small sampling area, the placement of the analyte deposit must be reproducible. This problem has been solved with the addition of a direct deposition system, which removes the sample thickness variability and positioning uncertainty of the deposit. The design consists of a series of values attached to a nebuliser. The values allow for the loading of the sample, nitrogen airflow, and cleaning of the nebuliser after deposition. To help contain the sample to a small area once deposition has taken place, there is a vacuum line attached to the nebuliser. This simple system allows for higher sensitivity and run-to-run reproducibility for minute sample deposits.

### Rapid screening of solid samples for mercury using pyrolysis option with a portable RA-915+ mercury analyser with Zeeman correction

*Michael Markelov, Sergey Sholupov, Joseph Siperstein, David Dechant and Olga Bershevits, Ohio Lumex Co., 5405 E. Schaaf Road, Cleveland, OH 44131, USA*

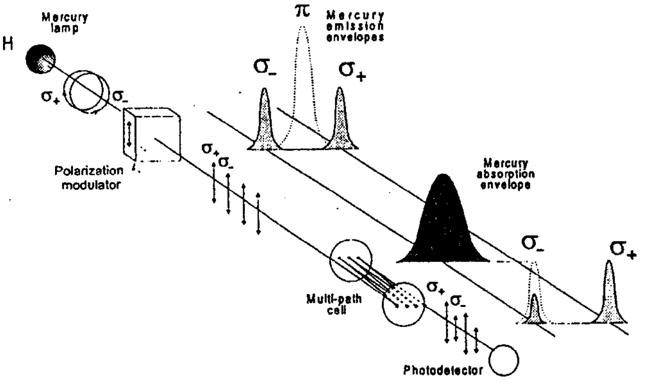

*Physical foundations and the operating principle of the RA-915+ mercury analyser.*

This paper will demonstrate a very simple technique for the rapid estimation of mercury content in solids, semisolids and liquid samples at ppb/ppm levels. The measurements take <1 min and <100 mg sample. The pyrolysis cell is easily attachable to a portable atomic absorption mercury analyser (RA-915+).

This instrument also possesses its own 10 m multipass cell for air monitoring at ppt levels. The selectivity of this method for mercury is greatly enhanced using Zeeman correction. It will be shown that the products of pyrolysis can easily be rerouted into this high sensitivity cell if needed. The repeatability of the technique is >10% RSD. This methodology was successfully used for the analysis of soils and polymers for mercury.

### Towards the simultaneous determination of AS and SE by hydride generation atomic absorption spectrometry

*David L. Scott and Julian F. Tyson, Department of Chemistry, University of Massachusetts, Amherst, MA 01003-9336, USA*

Oxidative sample digestions produce Se(VI) and As(V). Hydride generation (HG) requires Se(IV) and As(III); however, reagents that reduce As(V) to As(III) also reduce Se(VI) to Se(0), which is not borohydride active. It is known that bromide in acid solution is capable of reducing Se(VI) to Se(IV), and we have found that this reagent can also reduce As(V) to As(III). An unwanted side-reaction (the formation of arsenic tribromide) may be avoided by the addition of some bromate. Previous work has demonstrated the use of hydrobromic acid/bromate for the reduction to Se(IV) of organoselenium species in a microwave-assisted procedure. The mono- and dimethylated organo-arsenic species MMAA and DMAA have also been reduced to As(III) in an on-line microwave assisted procedure using the same hydrobromic acid/bromate reductant that reduces inorganic arsenic. The simultaneous on-line reduction of inorganic and organic species for both elements is thus possible, allowing the simultaneous determination of the elements in real samples. For the FI-hydride generation determination by atomic absorption spectrometry with quartz tube atomisation, the optimised borohydride concentrations for As and Se are quite different, reflecting the different roles of hydrogen in the atomisation processes. Graphite furnace atomisation with trapping on the iridium-coated interior was used to investigate the conditions for the simultaneous determination of the two elements. Interest in speciation studies prompted investigations into post-column application of the microwave-assisted procedure which would allow the detection of the elements after separation by HPLC. Results of attempts to implement the bromide/bromate reductions in the FI manifold will be presented.

### Electrochemical detection in conventional and chip-based non-aqueous capillary electrophoresis

*Ulli Backofen[1], Frank-Michael Matysik[2] and Craig E. Lunte[2], [1]University of Kansas, Department of Chemistry, Lawrence, KS 66045, USA, [2]Universitit Leipzig, Institut fur Analytische Chemie, Linnstr. 3, 04103 Leipzig, Germany*

Electrochemical detection in capillary electrophoresis (CE-EC) has been demonstrated to be an inherently sensitive detection principle for a variety of compounds including neurotransmitters, drugs and dyes. The strength of EC is that it can be easily miniaturised without compromising detection limits, requiring only a relatively simple and compact instrumentation. However, EC has not found general acceptance outside of electro-analytical research groups mainly because of (1) interference between the detection circuit and the high voltage field (HV), (2) difficult alignment of the sensing electrode with the separation channel, (3) EC's limited accessibility to compounds which are difficult to oxidise (reduce) and (4) occasionally poor reproducibility of the surface of the working electrode.

This presentation will demonstrate how these obstacles can be overcome by non-aqueous capillary electrophoresis (NACE) performed in fused silica capillaries and in the planar (chip) electrophoresis format. It has been shown that end-column detection, where the sensing electrode is placed in front of the capillary outlet, can be applied to NACE without incorporating any decoupler device. The potential offset at the working electrode, in the order of a few hundred millivolts, caused by the separation field can be easily compensated for by adjusting the voltage at the potentiostat. The extended potential window in organic media such as acetonitrile enables sensitive detection of amphetamines in acidic electrolytes and cannabinoids in strongly basic electrolytes at high potentials. Low detection limits in the ng $ml^{-1}$ range and highly reproducible peak currents were thus obtained. Furthermore, special attention has been paid to improving the signal–noise ratio by changing parameters affecting hydrodynamic characteristics of the detector such as the distance between the working electrode and the capillary outlet. The highest sensitivity was observed at a distance of 25 pm. The noise level at this distance was ~180 fA, which is only slightly higher than the electronic noise of the potentiostat. An optimised CE-EC set-up was applied to the determination of cannabinoids in hair samples after extracting the active compounds from the protein matrix.

Ongoing research is focused on the implementation of voltammetric electrodes in CE chips. Initial experiments are conducted using CE chips made from (poly)dimethylsiloxane (PDMS). The amperometric electrode is prepared in the chip by means of a combination of electroless metal plating and electroplating. Besides the inherent advantages of CE in the chip format, this approach circumvents the need for manual alignment of the working electrode with the separation capillary. The resulting device, including control units and power supply, should be compact and rugged, thus suitable for application in remote areas.

### Overview of single and multi-element mercury determination

*Zoe A. Grosser, PerkinElmer Instruments, 50 Danbury Road MS-219, Wilton, CT 06897, USA*

Mercury is a significant element because of its toxicity and because it may also show long-term cumulative effects at lower levels. It can bioaccumulate in the food chain, which is of particular concern for those who eat fish regularly. The number of fish advisories issued in the USA has increased over recent years, and these reflect fresh-water streams and lakes, although several marine advisories have been issued along the Gulf Coast. A recent EPA report stated that ~87% of the mercury emissions in the USA came from solid waste incineration and fossil fuel combustion. Mercury does not respect country boundaries because of its volatility. It is truly a global pollutant.

Mercury measurement techniques have advanced over the years from the exclusive use of cold-vapor atomic absorption to inclusion of multi-element techniques, such as ICP-OES and ICP-MS. The table summarises detection that can be seen for mercury determination with different techniques. This paper will summarise the capabilities of the techniques available for mercury measurement. Current regulations will be matched against the available analytical capabilities to aid in choosing the right technique.

*Mercury detection limits with atomic spectroscopy techniques.*

| *Technique* | *Preconcentration* | *Detector* | *Detection limit* ($ng\ l^{-1}$) |
|---|---|---|---|
| ICP-OES | none | OES | 8000 |
| ICP-MS | none | MS | 200 |
| Flow injection | none | AAS | 100 |
| Flow injection—continuous | Au/Pt gauze | AAS | 10 |
| Flow injection | none | FIMS | 4 |
| Flow injection—continuous | Au/Pt gauze | FIMS | 0.2 (20 ml) |
| Batch | Au/sand | AF | 0.2 (>100 ml) |
| Flow injection | graphite tube | AAS | 0.5 (100 ml) |
| Flow injection—continuous | Au/Pt gauze | ICP-MS | 0.2 (25 ml) |

### Perfecting the technique for the separation and speciation of six arsenic compounds present in human urine

*Lisa S. Milstein, Olujide T. Akinbo, Amal Essader, Edo D. Pellizari and Reshan A. Fernando, Research Triangle Institute, Analytical and Chemical Sciences, 3040 Cornwallis Road, Research Triangle Park, NC 27709, USA*

The toxicity of arsenic compounds is highly dependent on its chemical form: inorganic arsenicals being more toxic than organic arsenicals. Consequently, numerous investigations were performed to find a suitable mobile phase buffer for the speciation of arsenic compounds found in urine, using ion-exchange/inductively coupled plasma-mass spectrometry (IE/ICP-MS).

A Tris buffer mobile phase was initially evaluated for the urine samples because it was successfully used for the speciation of arsenic in drinking water samples. Only five peaks were visible using this mobile phase with the co-elution of AsB and As(III). Also, MMA and AsC peaks were not baseline separated. Since As(III) is one of the peaks that requires accurate quantitation, it was important to find a buffer system capable of separating all six arsenic components.

An ammonium carbonate buffer system was found to separate successfully all six arsenic species as well as an internal standard (potassium hexahydroxy antimonate (V)). No significant salt deposit was observed on the sampler and skimmer cones on the ICP-MS system; consequently, a high-throughput of samples could be evaluated.

A method performance evaluation for the speciation of arsenic in urine using ammonium carbonate buffer was determined. Estimating precision, linearity, detection limit and accuracy accessed analytical performance of the method.

Total arsenic content was determined as well using flow injection analysis (FIA) coupled to ICP-MS. Total arsenic results by FIA were compared with the sum of the arsenic species from the chromatographic analysis to verify that mass balance was achieved. The results of all these studies will be presented.

### Determination of inorganic and organic mercury by flow injection chemical vapor generation atomic absorption spectrometry

*Julian F. Tyson[1], Christopher D. Palmer[1] and Gerhard Schlemmer[2], [1]Department of Chemistry, University of Massachusetts, Amherst, MA 01003-9336, USA, [2]Bodenseewerk PerkinElmer, GmbH, Postfach 101164, D-88662 Uberlingen, Germany*

Many environmental and toxicological studies are underpinned by reliable information about the interconversion of inorganic and methyl mercury. Hence, there is considerable interest in the development of simple, rapid and reliable methods for the measurement of mercury species in a variety of sample materials. Existing methods are based on (1) the relative reactivity of the species towards borohydride and tin (II), (2) chromatographic separation of the ethylated species and (3) cryogenic trapping of various derivatives, including alkyls and methylmercury hydride. The detection limit for the measurement of inorganic mercury may be improved by the amalgam trapping of the mercury vapor, released from a relatively large sample volume, followed by pulsed thermal release. We have developed a speciation procedure based on the reactivity towards borohydride at low acid concentrations and the amalgam trapping of both mercury and methylmercury hydride. When the vapors are directly introduced in the absorption cell, only inorganic mercury is detected as methylmercury hydride does not absorb at 253.7 nm. The difference between the signal with amalgam trapping and that without amalgam trapping is interpreted as due to the methylmercury. Recoveries of 100% of both species from river, pond and tap water were obtained with detection limits of 10–20 ppt. Analysis of urine by standard additions is also possible. Accurate analyses of marine reference materials certified for total mercury and methyl mercury (DORM and DOLT from the NRCC) were obtained, though calibration was by standard additions to sample solutions in tetramethylammonium hydroxide. In contrast to other recently published findings, the Nation dryer did not interfere with the methylmercury determination, and we confirm that the reaction between borohydride and methylmercury does not produce significant amounts of mercury vapor; however, high concentrations of methyl mercury give an inorganic signal corresponding to 2–3% either of partial reaction or due to an impurity.

### Evaluation of liquid chromatography process control

*Lars Karlsson, Magnus Fransson, Anders Sparn and Bengt Lagerholm, Analytical Development, AstraZeneca R&D, S-431 83, MSIndal, Sweden*

In the pharmaceutical industry liquid chromatography (LC) is most often the technique of choice for the routine analysis of drugs and related substances. Typically, the only measure of system stability is standards, injected repeatedly throughout the sequence. In this paper we present a novel approach where the analytical sequence is treated as a *process* with the chromatographic data as the product of that process. Enhanced quality of the product, i.e. the chromatographic data and results derived thereof, can thus be obtained through monitoring the process, i.e. the chromatographic system.

For this purpose, the liquid chromatography process control (LCPC) system has been developed. Here, several system parameters (usually more than six), e.g. the pressure at the column and the injection valve, are continuously monitored. Chemometrics is used for interpreting the data and producing multivariate statistical process control (MSPC) charts. The chromatographic run is divided into two parts: the dynamic injection phase and the static elution phase. Two principal component analysis (PCA) models, one for each phase, are continuously created and upgraded as the data are collected.

In this paper, results from long-term testing of the system are presented, including assessment of the robustness, stability and need of calibration for the system sensors. The ability of the system to detect long-term drift is

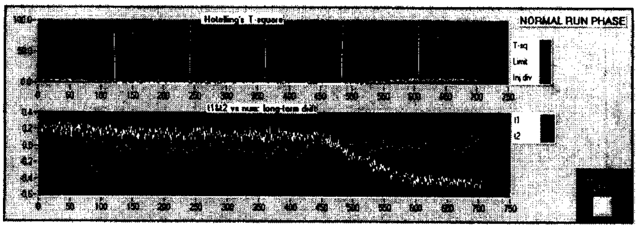

*Screen shot showing drift in the system.*

evaluated and the setting of appropriate error limits is discussed. It is shown that LCPC provides better control of the analysis, potentially allowing a reduction in the number of standards and replicates. Furthermore, troubleshooting is dramatically facilitated.

### Software solution for real-time decision-making intelligence for portable field sensors

*K. T. Lu, Dennis Baba and Thomas Leffler, Atomic Engineering Corp., PO Box 3342, Gaithersburg, MD* 20885, USA

Advances in technologies have promoted the development of many portable field sensors that require minimally invasive sample preparation and separation such as LIDAR, laser-induced breakdown (LIB) spectrometer and laser-induced fluorescence (LIF). Like all laboratory instruments, their analytical success depends on library data for internal calibration. The calibration problem is more serious for *in-situ* field sensors because of the lack of reliable library data for environmental matrix effects, such as soil matrix, moisture and atmospheric pressure etc. A critical need for the field sensor to achieve its potential as an *in-situ* analytical tool is the development of an extensive database of environmental matrix effects and the real-time decision-making intelligence.

We have developed a prototype software solution that is capable of integrating a vast amount of laboratory data and performing real-time decision-making intelligence. With this key technology solution, the users can integrate data from laboratory sensors with those *in-situ* field apparatus so that real-time calibration can be made using these data as references. This function is important because field conditions may change the laboratory-based calibration. The real-time decision-making intelligence is made possible by the efficient algorithms to perform fast analysis and concentration determination. This feature greatly enhances the performance of the field sensors.

We use RCRA trace elements in synthetic silicate and limestone, and stream sediments soil samples [1] measured by laser-induced breakdown spectrometer as an example to illustrate the power of the software. Because the sensitivity of the detection limit of a certain element is masked by the environmental matrix effect in the soil sample generated by the laser-induced plasma,

1. 'Certified Soil and Sediment Sample GBW Series: State Bureau of Technical Supervision, China' imported by Brammer Standard Comp., Houston, TX, and LIBS data were provided by David Cremers, Los Alamos National Laboratory, private communication.

it is essential to calibrate the detector with databases under field matrix condition. Two databases with the above mentioned data files as input are created using the software system. The database files are determined by calibrating the selected 'finger prints' of the analyte relative to AEC's reference database. These database files can then be loaded by the search program at another time to be compared against similar files to determine the detection limit to achieve real-time calibration.

The software system is capable of combining, integrating and analysing all these laboratory data, data from portable field detectors and databases. The efficient algorithms can carried out the data logging, searching, real-time calibration and decision-making at once in a few steps. The combination of improved software and the advanced algorithms make the real-time analysis of the trace elements in soil sample from portable field sensors possible.

### Interactive analytical chemistry on the Web

*James A. McQuay, Jillian Schickler, Darin Freitag, Alicia Gamblin and Vassili Karanassios, Department of Chemistry, University of Waterloo, Waterloo, Ontario, Canada N2L 3G1*

Thanks to the Internet, the World Wide Web has revolutionised the way we do things analytically. For example, the Web provides the capability to access archival information, databases, product catalogs and product specifications rapidly. It also offers the ability to search and access teaching aids and research publications world wide [1]. A key benefit of using a Web browser is its multimedia capabilities that stem from an inherent ability to mix text, graphics, video and audio using readily available software. An example is shown in the figure.

In this presentation, several of the unique capabilities of the Web will be demonstrated in the form of a video poster on a laptop computer [2]. Examples include course home pages, Web-executable programs, streaming videos that may be used for electronic teaching purposes (e-teaching), digital videos that may be used for training retraining purposes (e.g. an easy-to-assemble LEGO® monochromator [3]), QuickTime® virtual reality tours, and videos of an inductively coupled plasma-mass spectrometer (ICP-MS) and of a sample introduction system for ICP-AES (atomic emission spectrometry).

1. Karanassios, V., 2000, *ICP Info. News Lett.*, **26**, 152.
2. McQuay, J. A., and Karanassios, V., 1999, Pittsburgh Conference, paper 1970P.
3. Shickler, J., and Karanassios, V., 2000, Conference on Plasma Spectrochemistry, Ft Lauderdale, FL, paper ThP50.

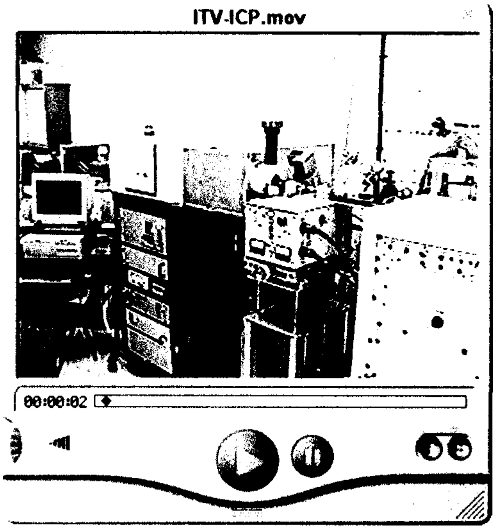

*QuickTime® movie of an ITV-ICP-AES system.*

### Role of chemometrics in chemical image analysis

*Robert C. Schweitzer, Arjun S. Bangalore, Scott A. Keitzer and Patrick J. Treado, ChemIcon, Inc., 7301 Penn Avenue, Pittsburgh, PA 15208, USA*

The need to determine the spatial distribution of chemical species in both manufactured and natural products is driving increasing numbers of companies to chemical imaging. Chemical imaging combines molecular spectroscopy and digital imaging for the chemical analysis of materials. We have developed a variety of chemical-imaging platforms to serve customer needs based on a number of spectroscopic techniques including Raman, NIR, UV-VIS, fluorescence, photoluminescence and polarised light. A critical component of chemical imaging systems is the software. The functionalities required to cover the entire analysis cycle illustrated in the figure include preprocessing, qualitative and quantitative mapping, feature recognition, and automation. Since chemical imaging combines molecular spectroscopy and digital imaging, all of the standard spectroscopic and related chemometric tools as well as the traditional digital image-processing tools are applicable. This presentation will explore several applications that illustrate the synergism resulting from the combined application of these two sets of analysis techniques.

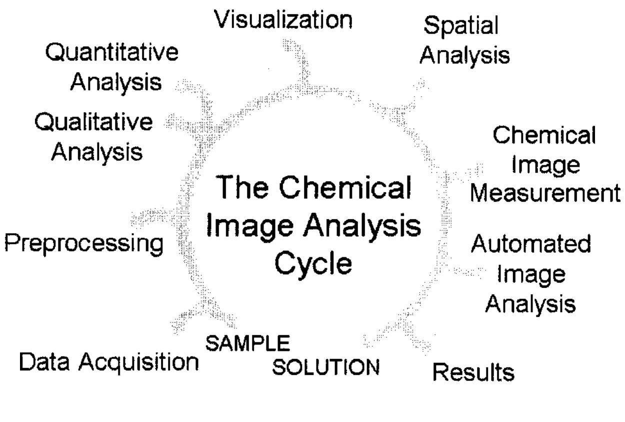

### Automated MS direct probe for use in an open-access environment

*Mark J. Hayward*[1] *and John J. Manure*[2], [1]*Novartis Pharmaceutical Corp., 556 Morris Avenue, Summit, NJ 07901,* [2]*Scientific Instrument Services, 1027 Old York Road, Ringoes, NJ 08551, USA*

An automated direct exposure probe (DEP) interfaced to a mass spectrometer has recently been developed and optimised for use in an open-access environment in a pharmaceutical development laboratory.

The automated DEP probe uses a platinum filament wire for the analysis of samples. Samples to be analysed are dissolved in a suitable solvent and are then injected onto the DEP filament wire using an automated syringe injector. The sample is introduced into the mass spectrometer (MS) and analysed. The PC software to control the automated probe is fully integrated into the MS software to provide for a seamless probe–MS operation. The result of this design is an automated MS direct probe that can analyse samples at 2–5 min per sample.

The automated probe was developed and integrated into an open-access environment in a pharmaceutical development laboratory. The open-access system permits inexperienced chemists and technicians to submit samples directly to the MS for analysis. For a sample to be analysed, the chemist fills out a simple form on a PC screen including the submitter's name and sample name. He then selects a method of analysis from one of several standard methods pre-established by the supervisor. After completing the form, the user is instructed where to place the sample. The samples are then analysed automatically and the results reported back to the chemist with little or no interaction of the MS operator. This results in increased productivity from the MS laboratory with less technical staff requirements.

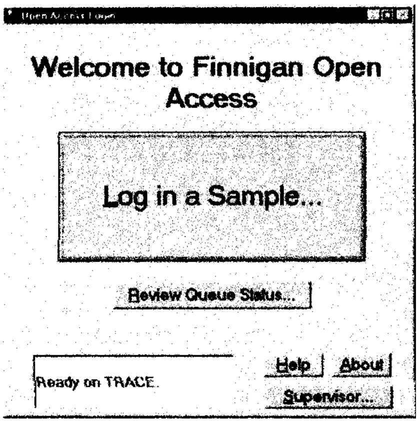

### Critical analysis of USEPA method 5035 using a robotic vial autosampler

*Glynda A. Smith, Eric T. Heggs and Mark Krigbaum, Tekmar-Dohrmann, 7143 East Kemper Road, Cincinnati, OH 45249, USA*

In today's laboratories, increased efficiency and productivity are of extreme importance. Equally important is the ability to automate analyses without sacrificing sample integrity or data quality. A vial autosampler has been developed to automate fully purge and trap analysis of water, wastewater, and solids samples in accordance with current USEPA methods for volatile analysis.

Research will demonstrate the use of a robotic vial autosampler with USEPA 5035 preparatory method for the analysis of USEPA Method 8260 analytes. The research will compare the data quality achieved with the robotic vial autosampler against results obtained for USEPA Method 8260 using other preparatory methods involving discrete autosamplers. The research will also provide analytical proof of the low carryover associated with running soil samples on the robotic system. All data will be evaluated for linearity, precision and accuracy.

### New automated multichannel pipetting workstation with variable span capability

*Gary Engelhart*[1] *and Joerg Pochert*[2], [1]*Hamilton Co., 4970 Energy Way, Reno, NV 89502, USA,* [2]*Hamilton Bonaduz AG, Via Crusch 8, CH-7402 Bonaduz, Switzerland*

At the beginning of 2001, Hamilton's new Microlab® STAR—Sequential Transfer and Aliquoting Robot—is to be launched in the USA. Microlab STAR is designed as a highly flexible pipetting workstation with medium-to-high throughput for pharmaceutical and genetics applications. The instrument is equipped with eight individually spreadable pipetting channels with random access to all deck positions, so as to guarantee maximum flexibility for the assay set-up. It runs under an easy-to-use Windows NT 4.0-based software, which handles the full flexibility of the hardware. Microlab STAR is the first fully automated multichannel instrument featuring the monitored air displacement (MAD) pipetting principle, without using any system liquid. In addition, the new CO-RE (compression-induced O-ring expansion) technology is designed to pick up disposable tips or reusable disposable steel needles during the same run and enables a very accurate positioning of the pipette tip and an aerosol-free tip release. All pipetting channels are equipped with sensors for capacitance- and pressure-based liquid level detection so as to detect the surfaces of both conductive and non-conductive liquids. Disposable tips or needles are available in three different sizes covering a volume range from 1 to 1000 μl with high accuracy. Microlab STAR processes all kinds of labware, from tubes to high-density microplates, including barcode identification. Relevant data from a variety of applications, such as drug screening, PCR preparation or DNA sequencing, will be presented.

### Low-cost automated microplate pipettor/dispenser

*Jason Greene and Michael Sevigny, Bio-Tek Instruments, Inc., PO Box 998, Winooski, VT 05404, USA*

Bio-Tek Instruments, Inc., has recently developed Precision 2000, a low-cost, automated liquid-handling system especially designed for microplates. This versatile device can transfer samples, fill plates in replicate, conduct serial dilutions, add reagent, mix within a well and continuously dispense reagent using a manifold. These operations may be carried out on both 96- and 384-well plates.

The system incorporates disposable tips that are automatically picked by a uniquely designed eight-channel pipette. Each of the eight channels can hold up to 120 gl of fluid per operation. Bio-Tek's proprietary pipette technology provides a reliable way to pick up and seal standard tips with controlled force. Each of the eight syringes is free floating within the body of the pipette. Precision 2000 solves the sealing issues associated with conventional pipettes. When tips are picked, the syringes are free to move up to accommodate differences in height and radially to accommodate deviations from ideal position. Precision 2000's unique XY transport design provides effortless 96- and 384-well plate transfers with the same pipette mechanism.

The configurable six-station platform allows for customised liquid transfers. Reagent troughs, tip boxes and up to six microplates can all be positioned where they best suit the customer's application. The entire platform can be removed for easy cleaning. Additionally, robotic plate handlers can be seamlessly interfaced to Precision 2000's multiple robot friendly stations via an available ActiveX TM component.

The goal of our R&D Department was to develop a versatile device that would meet the needs of individual technicians at an affordable cost. We have thus developed a system that can replace all of a laboratory's routine manual pipetting operations without being a tabletop behemoth. The device is small enough to fit inside a laboratory hood, thus allowing the use of hazardous reagents with minimal technician exposure. This, along with its lower cost, allows it to be utilised as a personnel workstation. Precision 2000 additionally helps eliminate repetitive motion injuries. Precision 2000 ensures fast, flexible and precise automated liquid delivery. Rapid dispense mode is made possible with an optional eight-channel manifold. A separate bidirectional syringe pump allows for the continuous rapid dispensing of reagent from any size reservoir to the six stations.

Precision 2000's onboard keypad and microprocessor can be utilised to program the steps required to conduct any operation. Precision Power$^{TM}$, a Windows-based PC software application, can be used to program complex plate operations quickly. Both the onboard microprocessor and the PC software provide ease of use by allowing the chaining of procedures. An intuitive program structure with several preprogrammed liquid transfer routines makes short work of the set-up. Specifications, operation, programming and features of the device will be described in the poster paper.

### New automated direct probe for a mass spectrometer

*John J. Manura and David J. Manura, Scientific Instrument Services, 1027 Old York Road, Ringoes, NJ 08551, USA*

The direct probe was one of the earliest techniques used to introduce samples into a mass spectrometer (MS). The technique is still popular today because it provides a means to perform rapid sample analysis with minimal or no sample preparation. However, the technique has been limited by the fact that it was not reproducible or quantitative. The direct probe was normally operated by trained users and did not lend itself for use in an open-access environment.

This study describes the design of a new automated direct exposure MS probe (DEP) that addresses these limitations. The DEP probe uses a platinum filament wire for the analysis of samples. Samples to be analysed are dissolved in a suitable solvent and then injected onto the DEP filament wire using an automated syringe injector. After injection, the sample solvent is evaporated from the wire using a small current. The sample is introduced through a vacuum isolation valve and into the MS where it is subsequently analysed. The system is completely automated and computer controlled. The software to control the automated probe is fully integrated into the MS software to provide for a seamless probe–MS operation package. The result of this design was an automated MS-direct probe that can analyse samples quantitatively at 2–5 min per sample. This paper describes the design of the system and demonstrates the analysis of analytical samples.

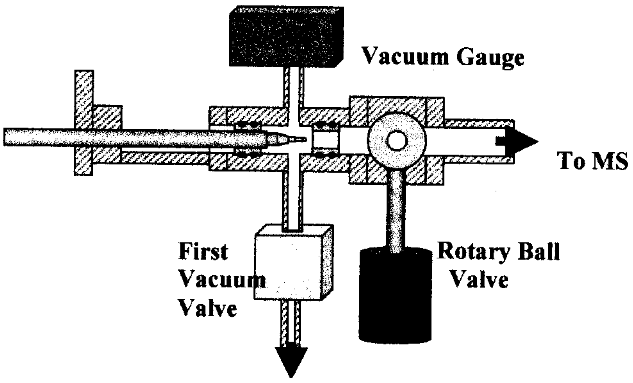

### Using an electronic nose to determine lipid oxidation

*Casey C. Grimm[1], Elaine T. Champagne[1] and Ed Bunge[2], [1]Food Processing & Sensory Quality, USDA-ARS-SRRC, 1100 Robert E. Lee Boulevard, New Orleans, LA 70124, [2]Nordic Sensor Technologies, One Exchange Place, Suite 1000, Jersey City, NJ 07302, USA*

Rice varieties, including waxy, non-waxy and aromatic, from different crop years were analysed. Milled rice (5 g) was placed in 20-ml vials and sealed with a screw cap equipped with a Teflon liner. Samples were stored at 5, 25 and 40 °C and sampled regularly over 3 months. The headspace was purged from the sample vial using purified air at 60 ml min$^{-1}$. The electronic nose was equipped with 10 MOSFET type and 12 MOS types sensors. Cycle times of 7 min were employed and raw data files generated. Rice samples were concurrently analysed by solid-phase micro-extraction GC/MS.

E-nose data were analysed using principal component analysis (PCA). Plotting of the primary component against the secondary resulted in readily discernible groups, with the primary component containing as much as 90% of the variability in the data set. Confidence in the analyses was obtained by comparing multiple vials containing the same sample against a single sample analysed multiple times. Standards of hexanal at concentrations of 10 and 100 ppm in water were used to correct for any instrumental drift. Both varietal differences and aging differences could be observed. However, varietal differences were small relative to aging.

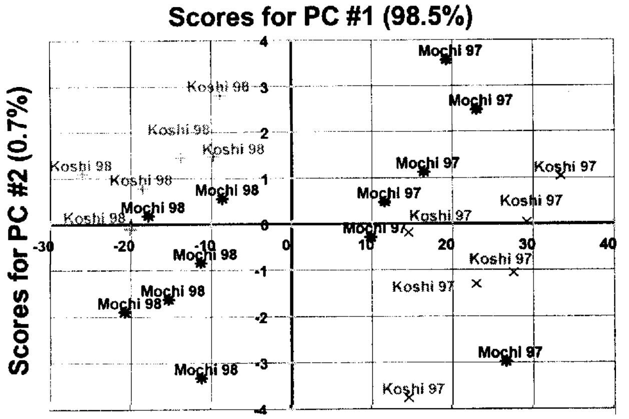

*PCA of two rice samples from two different years. Clear separation is observed between the 1997 and 1998 samples.*

### Development of a CEM for on-line speciation of mercury in flue gas

*W. T. Corns, P. B. Stockwell and D. W. Bryce, PS Analytical Ltd, Arthur House, Crayfields Industrial Estate, Main Road, Orpington BR5 3HP, UK*

Accurate measurement of mercury speciation in utility flue gas is necessary to model the fate and transportation of mercury in the atmosphere and to evaluate the effectiveness of mercury control technologies. Impinger-based methods such as EPA methods 29 and 101A have been successfully applied to determine total mercury. Modifications to those methods have allowed differential speciation for elemental and ionic forms of mercury. However, the results to date have raised more questions than answers. There is clearly a need within the industry to monitor mercury emissions continuously. A reliable approach using atomic fluorescence spectrometry has been developed and will be described.

The CEM developed uses a dual-stage purge-and-trap system based on amalgamation atomic fluorescence spectrometry at elevated temperatures. A sample interface to distinguish between elemental and total mercury has been developed thus enabling speciation of ionic mercury by difference. Results will be presented for both pilot- and bench-scale studies. The effects of various flue gas composites on the accuracy of the measurements will also be discussed.

The fully automated system developed has been evaluated on sites both in Europe and the USA. Results obtained compared well with the standard methods. The system performed consistently and mirrored the variations found in the standard methods with a significant reduction in the time-scales needed for the standard methods. Results will be presented showing the current status of the evaluation trials.

### Real-time monitor for ultratrace levels of ammonia and volatile amines in clean room ambient air

*Arnold E. Williams, Inga Henderson and Rebecca Gammill, Timberline Instruments, Inc., 1880 S. Flatiron Court Suite H-2, Boulder, CO 80021, USA*

A gas diffusion conductivity detection system has been developed to monitor ammonia and volatile amines in ultratrace levels in semiconductor fabrication clean room ambient air. Ammonia/amine levels as low at 10 ppb have been shown to effect adversely the production of semiconductors using deep UV radiation with chemically amplified photoresist lithography.

The system uses a microporous membrane that allows the ammonia to migrate into a very dilute, neutral pH buffer and reabsorb as ammonium. The adsorbed ammonium increases the conductance of the buffer. The buffer conductivity is measured with a highly stable ultrasensitive temperature-controlled detector. The detector response is proportional to the ammonia present in the original sample.

The accuracy of the system will be confirmed by calibration with a certified gas blend and permeation tube. This new instrument is capable of measuring a discrete air sample containing 1–50 ng ammonia in $<1$ min with reproducibility $>5\%$. With a continuous air-sampling system, it is possible to detect 0.5 ppb in a 5 SLPM air stream.

The system will require minimal maintenance, uses only easy-to-dispose dilute aqueous reagents and requires only minimal attendance.

### Speciation of selenium compounds using liquid chromatography particle beam/hollow cathode atomic emission spectroscopy (LC-PB/HC-AES)

*Jennifer L. Robichaud, W. Clay Davis, Melissa A. Dempster and R. Kenneth Marcus, Department of Chemistry, Clemson University, Clemson, SC 29634-0973, USA*

A system for the separation and detection of inorganic and organic selenium compounds utilising particle beam-hollow cathode glow discharge atomic emission spectroscopy (PB/HC-AES) as a selenium-specific detector has been investigated. Using LC-PB/HC-AES, the following compounds were examined: selenocysteine, selenomethionine, selenoethionine, sodium selenate and sodium selenite. A reverse-phase ion-pairing chromatographic method was developed to separate these five compounds, with UV absorbance monitored at 210 rim. The Se(I) 204.0 nm emission intensity was then monitored for the corresponding peaks by coupling the LC column with the PB/HC-AES system. The PB interface includes a thermoconcentric nebuliser to generate a finely dispersed aerosol, a heated metal spray chamber for desolvation and a two-stage momentum separator that removes solvent vapor. The resulting beam of dry analyte particles is introduced into a heated (~250 °C) hollow cathode, where they are vaporised, atomised and excited within the plasma. The retention times of the five analyte peaks are very similar to those detected by UV absorbance, demonstrating the ability of the PB interface to preserve the chromatographic integrity of the separation. Liquid chromatographic separations of the selenium-containing compounds demonstrate the feasibility of the PB/HC-AES system as a selenium-specific detector for liquid chromatography.

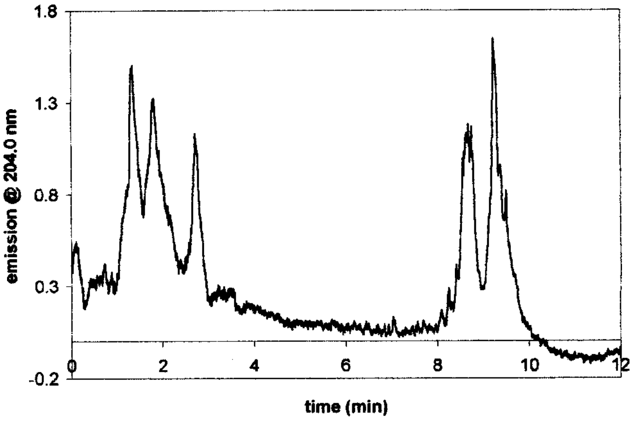

*Separation of 2.0E-03 M selenium compounds using reverse-phase HPLC with PB/HC-AES detection at 204.0 nm.*

### Arsenic speciation in blood of living rats by on-line microdialysis/microbore-HPLC/inductively coupled plasma mass spectrometry hyphenated system

*Wei-Chang Tseng, Tai-Ling Hsia, Yuh-Chang Sun and Mo-Hsiung Yang, Department of Nuclear Science, National Tsing-Hua University, Hsinchu, Taiwan*

To investigate the biological behavior of arsenic species in blood, some analytical difficulties including the extremely low concentration of interesting species, the high level and complicated sample matrix and small sample size (μl) will inevitably be encountered. In the present study, a hyphenated system consisting of microdialysis sampling, microbore-HPLC separation and inductively coupled plasma mass spectrometry (ICP-MS) on-line detection is developed to determine four arsenic species—arsenite (AsIII), arsenate (As(V)), monomethylarsonic acid (MMA) and dimethylarsinic acid (DMA)—in blood.

To accomplish the on-line analysis, an on-line injector was employed as an interface allowing the connection of microdialysis and microbore-HPLC. Microdialysis sampling was conducted by using saline solution (0.9% NaCI) as a perfusate and the dialysate at flow rate of 0.5 μl min$^{-1}$ was led to a 5 μl collection loop for further injection to HPLC for separation. The microdialysis probe consisted of a metal-free dialysis membrane of polycarbonate and had respectively a length and diameter of 10 and 0.5 mm. It had a high recovery and least contamination. For arsenic speciation by HPLC, a packed microbore anion-exchange column (1.0 × 150 mm, Nucleosil SB 100-5) was employed. Chromatographic separation conditions such as pH, concentration and flow rate of the mobile phase were optimised based on mutual requirements of chromatographic performance and detection sensitivity. The optimal separation was achieved with the use of pH 5.4 and 20 mM phosphate buffer solution and at the flow rate of 80–100 μl min$^{-1}$. With the established system, the four arsenic species can be completely separated within 10 rain and the limits of detection of 1.6, 4.2, 1.7, 1.9 ng ml$^{-1}$ for As(III), As(V), MMA, DMA can be respectively achieved. Also, the spectral interferences caused by the abundant chloride ions in microdialysate in ICP-MS measurement can be completely eliminated via this prior separation step. The simultaneous determination of arsenic species was subsequently carried out by coupling the microbore column with a microconcentric nebuliser (MCN) and ICP-MS. The performance characteristics of the proposed on-line microdialysis/microbore-HPLC/ICP-MS system are discussed in detail here. The practical applicability of the method has been tested for the analysis of arsenic species in the blood of living rats.

### Flow injection analysis of hydrogen peroxide and glucose based on copper-plated screen-printed electrodes

*Jyh-Myng Zen, Hsieh-Hsun Chung and Annamalai Senthil Kumar, Department of Chemistry, National Chung-Hsing University, Taichung 402, Taiwan*

A disposable copper-plated screen-printed carbon electrode (CuSPE) was developed for the determination of hydrogen peroxide by flow injection analysis (FIA) at ambient temperature without deoxygenating. Cyclic voltammetry on the CuSPE in pH 7.4 phosphate buffer solution showed the growth of CuO and Cu20. A well-defined reduction signal corresponding to the mediation of CuO and Cu20 occurred in the presence of hydrogen peroxide. The mechanistic study revealed that reduction is a coupled-chemical reaction mechanism with the operation of *pseudo-first order kinetics* on the concentration of Cu20. The calculated electrochemical rate constant ($k_e$) from Laviron model is 13.7 s$^{-1}$.

Systematic investigations were made to optimise the experimental parameters for hydrogen peroxide detection by FIA. With a poised potential of −0.3 V versus Ag/AgCI and a flow rate of 2 ml min$^{-1}$, the calibration curve was linear up to 200 pM $H_2O_2$ with a detection limit of 0.97 μM ($S/N = 3$). An enzyme reactor coupled with the CuSPE was further developed for glucose monitoring. The electrocatalytic reduction of enzymatically produced $H_2O_2$ at the CuSPE was determined by FIA in pH 7.4 PBS. Under the optimised conditions in FIA, the current signals were linear in the range 10–300 pm glucose with a slope and detection limit ($S/N = 3$) of 0.0114 pA pM$^{-1}$ and 1.94 pM, respectively. The RSD for 10 continuous injection of 10 μM glucose is 3.77%. Interference from ascorbic acid, dopamine, uric acid and acetaminophen showed a tolerable effect at equal molar concentration. The proposed method was applied to determine glucose content in fruit juice and clinical sample. Satisfactory results with good recoveries were obtained.

### Determination of trace elements in forensic bullet samples by plasma source mass spectrometry—2. Removal of lead by flow injection precipitation and filtration of sulfate

*Brian C. Weitze, Emily R. Yourd and Julian F. Tyson, Department of Chemistry, Massachusetts, Amherst, MA 01003-9336, USA*

The determination of the traces of Ag, As, Bi, Cd, Cu, Sb and Sn helps link recovered bullets with suspected crime weapons. The high concentration of lead makes the determinations by ICP-MS difficult, and a method to remove the lead by precipitation as lead sulfate has been developed. In a batch process, the lead concentration (as monitored by flame atomic absorption spectrometry) was decreased from 12 900 to ~6 ppm by reaction with 5% sulfuric acid and filtration through a 0.45-μm filter. A flow injection manifold was designed to implement the mixing, precipitation and filtration steps. The sample was injected into a 2% nitric acid carrier and merged with 5% sulfuric acid. The precipitate was retained on a down-stream filter while the analytes proceeded to the detector. After optimisation of the residence time, temperature and filtration medium, samples containing up to 12 900 ppm could be processed. Reagents for dissolving lead sulfate and cleaning the filters were examined. The best candidate was the trisodium salt of *N*-hydroxyethylethylene-diaminetriacetic acid. Determination of Cu and Sb (also by atomic absorption spectrometry) showed minimal retention of these analytes. The manifold was applied to the determination of the remaining elements by ICP-MS in NIST SRMs 2425 (battery lead), 2416 (bullet lead) and 2417 (lead-base alloy).

### Process optimisation procedure for multivariate optical element (MOE) manufacture

*Olusola O. Soyemi[1], Lixia Zhang[1], Paul J. Gemperline[2], Hong Li[1], Delyle Eastwood[1] and Michael L. Myrick[1], [1]Department of Chemistry and Biochemistry, University of South Carolina, 631 Sumter Street, Columbia, SC 29208, [2]Department of Chemistry, East Carolina University, Greenville, NC 27258, USA*

The use of optical coatings as computation devices for predictive spectroscopy has previously been described [1]. Optical computing provides a viable means of chemical analysis using simple hand-held devices with no moving parts. The main challenge in the manufacture of these devices is the design and manufacture of the interference filters. A novel filter design technique has been developed that creates a design which produces a structure that gives the best prediction error for the analysed species. Manufacture of the interference coatings is carried out via magnetron sputtering in which alternate layers of a high index material and a low index material are coated on a suitable substrate according to a specified filter design.

However, it is quite difficult, if not impossible, to deposit the multilayers accurately in the prescribed fashion due to manufacturing errors inherent in the deposition process. Since the overall success of the optical computing device depends on the ability to manufacture filters that have transmission profiles that match the desired structure as accurately as possible, this presents a real problem. To monitor the deposition process adequately, a method of continuous synthesis of filter coefficients (i.e. layer thicknesses) that adjusts the filter design on-line to account for the changing manufacturing conditions using custom-designed software is described. A comparison of filters manufactured from this method of 'dynamic' design, with filters manufactured from a single initial design, will be made.

1. Myrick *et al.*, 1998, *Anal. Chem.*, **70**, 73.

### Automated development of HPLC methods

*S. Galushko[1], I. Shishkinai[2], V. Tanchuk[2] and W. D. Beinert[3], [1]Im Wiesengrund 49-b, 64367 Muehltal, Germany, [2]Institute of Bioorganic Chemistry and Petrochemistry, National Academy of Science of the Ukraine, Kiev, Ukraine, [3]Merck KGaA, Frankfurter Str. 250, Darmstadt, Germany*

The objective was to study an applicability program with an artificial intelligence module development of HPLC methods of a new computer for fully automated ChromSword-Auto (Merck KGaA, Darmstadt, Germany), a real-time expert system for the automated method development which runs on-line with the Merck–Hitachi Lachrom2000 HPLC system. The system interacts on-line with the HPLC system to get data and update its methods. It can test several solvents and columns.

Mixtures of sweeteners, preservatives, sulfonamides, polyaromatic hydrocarbons, phenols, aromatic amines, due staffs, $\beta$-blockers, etc. that cover a wide range of compound properties have been used to test for unattended method development. Several scenarios have been tested: (1) the development of isocratic methods, (2) the development of gradient methods, (3) the optimisation of temperature, (4) to start from structural formulae, (5) to start from an arbitrary concentration of an organic solvent, and (6) in performing multi-experiment optimisations with different columns and solvents. The system finds the initial conditions from the structural formulae of compounds to be separated and the type of RP column to be used, or starts optimisation from arbitrary conditions when the structural data are unavailable. During optimisation, the self-learning system monitors data and searches for optimal isocratic or gradient conditions.

The results of automated optimisation with conventional trial-and-error and computer-assisted methods have been compared. In many cases, unattended automated optimisation led to better conditions (less analysis time, simple gradient profile, better resolution) than a trial-and-error procedure and it required much less human resources and user qualification then the computer-assisted method development.

### Automated analysis of DNA fragments in microchip electrophoresis systems

*Steven M. Wishnies[2], Akihiro Arai[1], Hiroshi Tanaka[1], Yoshiaki Aso[1], Hirohisa Abe[1], Shuzo Maruyama[1] and Teruhisa Ueda[1], [1]Shimadzu Corp., 1 Nishinokyo-kuwabaracho Nakagyo-ku, Kyoto 604-8511 Japan, [2]Shimadzu Scientific Instruments, Inc., 7102 Riverwood Drive, Columbia, MD 21046, USA*

Microfabricated or chip (microchip) electrophoresis has achieved remarkably rapid development, and instrumentation continues to advance toward a fully automated tool. One of the applications in which microchip electrophoresis systems have had the biggest impact is in DNA separations, in large part because microchip electrophoresis offers some clear advantages over slab gel electrophoresis for automation, speed and quantitative capability. The key characteristic required for the instrument in this regard is a PCR tube/plate compatible autosampler that might be consecutively accessible after amplification and desalting. Second, a liquid-handling unit for filling the channels with buffer and for loading the sample should be equipped with some flexibility depending on the channel design used, even when a high-viscosity polymer sieving matrix should be applied. Third, automated data processing of fragment size and quantitation with slab gel pattern is usually convenient for actual analysis.

This work presents a fully automated microchip electrophoresis system with a microtiter plate compatible autosampler and a novel linear-imaging UV detector in which the entire region of the separation channel is monitored using a 1024-element photodiode. The analytical conditions are optimised to give the best separation of DNA fragments. Examples of PCR products will also be demonstrated.

### Difficult matrix introduction: a new way of automating trace analysis in complex matrices

*Peter Tablack[1], Sjaak De Koning[2], Diane Nicholas[3] and Ray Perkins[3], [1]ATAS Deutschland, Beisterweg 13, D-44227 Dortmund, Germany, [2]ATAS International, De Run 4441, 5503 LS, Veldhoven, The Netherlands, [3]ATAS UK, Broadway House, 149–151 St Neots Road, Hardwick CB3 7QJ, UK*

Difficult matrix introduction (DMI) is a technique that uses a direct thermal desorption (DTD) instrument for the handling of numerous difficult samples in gas chromatography. A small, low-cost glass vial is inserted into the DTD liner, which is placed into the injector by a robotic sampler. Here, the sample is thermally desorbed. On removal, the vial is disposed of, whereas the liner can be kept for reuse.

There are a wide range of sample matrices that may be introduced in this way, including solids, e.g. food, powders, slurries, aqueous samples, and dirty or reactive samples. The liner, column and detector, particularly MSDs, are protected from the heavy, non-volatile components of the sample by retaining them in the insert, while transferring the more volatile components of interest. This is achieved by optimising the final injector temperature necessary for this selective transfer.

Various applications of the technique are discussed including the following.

- Pesticides in fruit juices.
- Silicone contamination in paint.
- Fragrances in soap.
- Nicotine in tobacco.

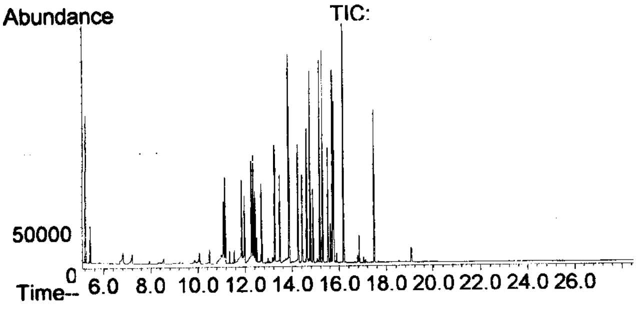

*Automated analysis of [1]HI ethyl acetate extract of apple juice spiked with pesticide standard mix. Extract run without clean-up.*

### Automatic contact pressure system for ATR applications in FTIR spectroscopic accessories

*Joseph P. Lucania[1], David W. Schiering[2] and Gregg Ressler[3], [1]Harrick Scientific Corp., PO Box 1288, Ossining, NY 10562, [2]SenslR, 15 Great Pasture Road, Danbury, CT 06810, [3]Spectra-Tech, Inc., PO Box 869, Shelton, CT 06484, USA*

A new electromechanical design is described that allows controlled pressures to be applied to samples in ATR accessories. Previous devices developed by accessory manufacturers used strictly mechanical equipment to accomplish this task. The new design uses a reversible DC motor, controlled by interface electronics and coupled to a rack-and-pinion mechanism. The stall current of the motor is limited to produce the desired pressure on the sample. Only downward pressure is exerted without any twisting movement.

The apparatus consists of a mechanical module and an electrical module, connected by a single electrical cable. The mechanical module contains the rack-and-pinion assembly mounted on a linear ball slide. The bottom end of the rack is used to contact the sample. The top end is blocked by a mechanical stop. The length of travel is $\pm 20$ mm. The pinion gear, which turns to move the rack, is incorporated on one end of a shaft assembly, which is supported on two pillars with radial ball-bearings. On the other side of the shaft is a knob for turning the mechanism manually. A small sprocket gear is centrally located on the shaft. This gear supports a gear drive chain, which is turned by a large sprocket gear mounted to the shaft of the motor. A torque reduction results. Tabs at the top and bottom ends of the rack are used to activate slotted optical sensors. The tabs and sensors define ranges, near the sample and the top mechanical stop, where a slower motor speed is used.

The electrical module contains interface circuitry that controls the motor speed, direction and current. A display of the motor current, proportional to the pressure, is given. LEDs indicate when the mechanism has reached the bottom (sample) or top position. A fixed selection of a number of different current limits is provided. Hence, the pressure exerted on the sample can be readily set to an optimum and repeatable level. Once the sample pressure is set, the pressure cycle is initiated by throwing an electrical switch. To release the sample, the mechanism is raised by throwing the switch in the opposite direction.

### ASA-automated speciation analyser: easy and fast analysis from sample preparation via chromatographic separation to spectrometric detection

*Eike Kleine-Benne[1], Bernd Rose[1], Wolfgang Buscher[2] and Jorg Bettmer[3], [1]Gerstel GmbH & Co., KG, Aktienstra. 232-234, 45473 Mannheim an der Ruhr, [2]ICB—Institut fur Chemo-u. Biosensorik, Mendelstrasse 7, 48149 Munster, [3]Johannes Gutenberg-Universitat Mainz, Institut fur Anorganische u. Analytische Chemie, Duesbergweg 10–14, 55099 Mainz, Germany*

The quantitative determination of heavy metals in their individual chemical forms so called 'speciation' is possible but, because of the high equipment costs involved, is carried out only in a few laboratories. Speciation is now fast and easy thanks to the compact Automated Speciation Analyser (ASA) developed by the ICB (Institut fur Chemo-u. Biosensorik) within the framework of an EU project. The equipment prototype was developed and produced by GERSTEL GmbH & Co., KG. A technique is now available that enables chromatographic separation and spectrometric detection of individual organometal compounds within a few minutes. Automation allows a high degree of analytical accuracy and reproducibility to be obtained. For the first time ever, standardization of speciation analysis and interlaboratory comparability of values recorded (e.g. in soil samples) is now possible. For mercury species, the limits of detection are $<50\,\mathrm{ng\,l^{-1}}$. An integral part of the system is the sample preparation unit in which immediately after weighing the alkylation can be carried out. With a purge gas jet, the derivatized compounds can be transferred into a cooling trap via a drying unit. The advantages of this procedure are the effective derivatization and separation of the disruptive matrix without any loss on degradation. After a fast gas-chromatographic separation using a multicapillary column (MCC, Alltech), the species are detected by microwave-induced atmospheric helium plasma (MI P) at an output of 60 W. The element emission lines are detected by means of a plasma emission detector (PED) patented by ICB. The total system is still a prototype but preparations are being made for its introduction into the market.

### Determination of mercury in coal using vapour generation atomic fluorescence spectrometry

*D. W. Bryce, W. T. Corns and P. B. Stockwell, PS Analytical Ltd, Arthur House, Crayfields Industrial Estate, Main Road, Orpington BR5 3HP, UK*

The vast majority of mercury in the environment originates from anthropogenic sources. In the USA alone, 52 tons of mercury per year are released into the atmosphere from coal-fired power stations. In an attempt to reduce emissions into the environment, new regulations have been introduced in many countries, including the USA, requiring all coal-fired power stations >420 MW to monitor their mercury in coal on a regular basis.

Vapour generation atomic fluorescence spectrometry is an excellent technique for the determination of mercury with limits of detection <1 ppt, linearity over seven orders of magnitude and is relatively free from interferences. Detailed descriptions of a fully automated system for the determination of mercury based on continuous flow vapour generation atomic fluorescence spectrometry will be given. The instrumental and chemical conditions along with digestion procedures and results for both coal and coal ash certified reference materials will be presented.

An added bonus with this analytical approach is that the instrumentation can be used to analyse other material for mercury and the digest itself can form the necessary input for other trace elemental techniques.

### Remote chemical inspection of materials using Raman fiberscopes

*Ryan D. Smith, Scott A. Keitzer and Patrick J. Treado, ChemIcon, Inc., 7301 Penn Avenue, Pittsburgh, PA 15208, USA*

Raman chemical imaging is a powerful analytical tool that provides both chemical and spatial information about a sample. This technology will continue to benefit more fields as Raman imaging instruments become more compact and more portable. The use of fiberoptic systems to deliver and collect light is one way to increase the mobility of Raman chemical imaging instruments. The ChemIcon Raven Raman fiberscope is equipped with both laser light and white light illumination fibers and a coherent fiber bundle for spectral and image collection. Filters are incorporated in the collection end of the fiberscope to reject sufficiently Raman scatter generated in the laser delivery fiber and to reject adequately laser light to allow for the acquisition of Raman spectra and Raman chemical images from solutions and solids to within $200\,\mathrm{cm^{-1}}$ of the laser line. Raman chemical imaging is performed with the Raven by coupling it to an imaging spectrometer-based detection system. Raven fiberscopes have been designed to accommodate a variety of laser excitation wavelengths, making this technology applicable to a wide range of sample types. Results from several applications utilising the Raven will be presented and discussed.

### New instrumentation for automated high-speed bio-analysis

*Otto Halmingh, Dries Vrielink, Gerard Haak and Bert Ooms, Spark Holland BV, PO Box 288, 7800 AJ Emmen, The Netherlands*

On-line SPE-LC-MS is a well-accepted technology in the drug-development laboratory for automated assays of drugs in biological samples. Major merits include total automation, high precision and high sensitivity. For on-line SPE, the challenge is to keep pace with the LC-MS analysis cycle time to avoid waiting time for the (costly)

LC-MS. Ideally, the SPE part of the on-line SPE-LC-MS assay should not cause any LC-MS waiting time at all! Zero LC-MS waiting time for on-line SPE can be accomplished if SPE is faster than the LC-MS analysis and runs in parallel with the LC-MS run. Another major challenge in the drug development laboratory is the increasing number of assays that need to be developed for the increasing number of drug candidates that enter the preclinical phase.

We present new instrumentation for on-line SPE-LC-MS that has been designed to achieve assay cycle times of <1 min. Dedicated configurations allow for rapid method development with programmable change of all SPE parameters, including SPE temperature. A generic protocol has been developed to provide a successful start, with easy further optimization. Examples of high-throughput SPE-LC-MS and fast-method development will be presented.

### Automated in-syringe dilution of concentrated headspace samples and standards

*Ingo Christ and Edward Pfannkoch, Gerstel, Inc., 1510 Caton Center Drive, Suite H, Baltimore, MD 21227, USA*

Headspace GC is a widely used technique for the analysis of volatile and semivolatile compounds above solid and liquid samples, particularly when the sample matrix can interfere with the analysis. Autosamplers are available that can automate most steps of the analysis, from sample heating and agitation to heating the syringe used for sampling before injection.

Automated methods are usually designed to handle analyte concentrations within a reasonably limited range, most often one to two orders of magnitude. For some applications, sample concentrations may vary by three orders of magnitude or more, thus presenting a challenge to maintain chromatographic peaks on scale without altering split ratios and thereby the calibration of the system. Also, some samples contain multiple analytes in greatly disparate concentrations, usually requiring the analyst to prepare two or more dilutions of each sample before beginning the analysis. It would be convenient to automate headspace sample dilution to address these situations.

Calibration of the headspace GC usually requires preparation of several standards at different concentrations. Depending on the sample type, these standards may be more easily prepared in gas bags rather than sample vials, making it difficult to perform the initial calibration and any recalibration of the system using the same autosampler conditions used for sample analysis. Here again, it would be convenient to prepare a single concentration standard and automate the dilution immediately before injection.

We have developed a strategy and supporting software to automate headspace sample dilution using a commercially available autosampler. Samples or standards are drawn up into the heated headspace syringe and dynamically diluted with either air or clean inert gas. Examples of calibration curves generated using this in-syringe dilution method are given. Practical application of this technique for dilution of highly concentrated headspace samples is also shown. Accuracy and reproducibility of this automated dilution technique compared favorably with manual methods.

### HPLC method development with the aid of an automated pipetting and injection workstation

*Susan C. Dawson and Dan Lee, Hamilton Co., 4970 Energy Way, Reno, NV 89509, USA*

HPLC method development often involves repeated preparation of stock solutions, tedious dilutions of samples, hours sitting at the chromatography workstation waiting for the next injection opportunity, and the lost productivity of not working nights and weekends. Of course, there are autosamplers that will take your prepared samples and inject them without human attendance, but the samples must be made in advance nonetheless.

This paper explores the ways a robotic liquid-handling workstation can be used to ease the HPLC method development tedium. Hamilton Co.'s MICROLAB® 4000—equipped with an HPLC injector valve—was used to evaluate a new line of silica-based columns. On-the-fly dilutions and partial loop filling were employed to determine linearity, dynamic range, capacity, selectivity, efficiency and column life. The MICROLAB 4000 was electronically connected to the gradient pump as well as to the chromatographic data system's interface box. Coordinated programming of the instrument, the pump and the data system's controlling software enabled flexible, long-running performance studies. The capabilities of the coordinated system as well as chromatographic results will be presented.

### Blood serum profiling to track immune response using MALDI/TOF mass spectrometry

*Anthony M. Haag[1], Joseph Chaiban[2], Kenneth H. Johnson[3] and Richard B. Cole[3], [1]Department of Chemistry, University of New Orleans, Lakefront, New Orleans, LA 70148, [2]Core Labs and [3]Department of Microbiology, Immunology and Parasitology, Louisiana State University Medical Center, 1901 Perdido Street, New Orleans, LA 70112, USA*

Monitoring the immune response system of a host in response to an antigen is important in determining the type and severity of an infection. In this study, the use of matrix-assisted laser desorption/ionisation time-of-flight (MALDI/TOF) mass spectrometry has been developed as a tool for monitoring the immune response to bacterial infection. When employing the MALDI/TOF approach, the levels of many components of the blood serum have been found to be immune response-independent while others have been found to correlate directly with the response of the immune system to two known types of bacteria (*S. aureus*, *E. faecalis*). The relative intensities of blood serum peaks appearing at $m/z$ 14 966 and 8725 reproducibly changed in intensities as the infection progressed. The figure shows is the intensity ratio of $m/z$ 14 966 to 8725 as a function of time after infection was initiated. Each curve corresponds to a single mouse. The control group (bottom two curves, injected with phosphate buffer solution only) exhibited a nearly constant ratio over time, indicating no influence of exposure to buffer. However, mice injected with bacteria showed a reproducible increase in the *m/z* 14 966 to 8725 ratio over time. The increasing ratio may then serve as an indicator that the mice were developing an immune response. The relative amounts of the proteins yielding these peaks are thus useful markers of the progress of the immune response.

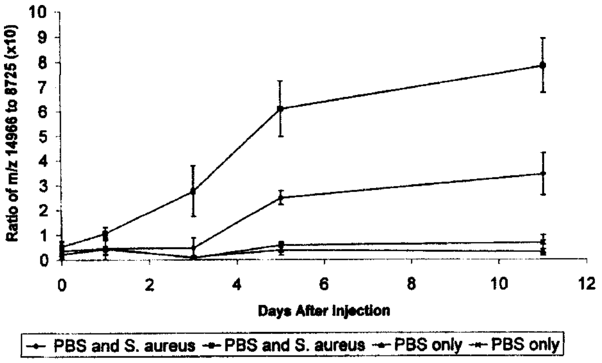

*Intensity ratios of m/z 14 966 to m/z 8725 versus days from beginning of infection.*

### Advances in IR database technology

*Michael Boruta, Bio-Rad Sadtler Division, 3316 Spring Garden Street, Philadelphia, PA 19104, USA*

IR database technology has vastly improved over the years with the advent of increasingly powerful hardware and software platforms. The increased power of these platforms has made possible many significant improvements in IR databases. These improvements have led to increases in both the resolution of the spectral data that can be effectively stored and in the amount and types of data that can be stored with each spectrum. This paper reviews these advances and predicts what the future of IR database technology may hold for spectroscopists and chemists.

### Automated control and operation of an FT-NIR analyser

*Edgar J. Magnuson, Leza M. Luchetta and Mark S. Kemper, Thermo Nicolet Corp., 5225 Verona Road, Madison, WI 53711, USA*

The inherent speed of analysis of Fourier transform near-infrared (FT-NIR) spectroscopy has greatly contributed to the techniques popularity, but the time to acquire a spectrum has made other aspects of the process seem slow by comparison. Consequently, users are interested in reducing complications related to sample presentation and the collection of appropriate backgrounds for references. Furthermore, validating a method using certified standards has become increasingly important in heavily regulated environments such as the pharmaceutical industry. An analyser that automates these essential tasks is highly advantageous because it makes analyses faster and more reliable while providing system performance verification at any time.

Another significant advantage of automating these functions is eliminating operator errors. Automation allows appropriate calibrated attenuation, detector gain and optical path to be selected without human intervention. The importance of automatic background collection with the sample in place will be discussed as well as how this automation improves spectral quality, decreases analysis time and reduces technician errors. Examples of automatic generation of collection of spectra and data analysis of certified standards will also be shown.

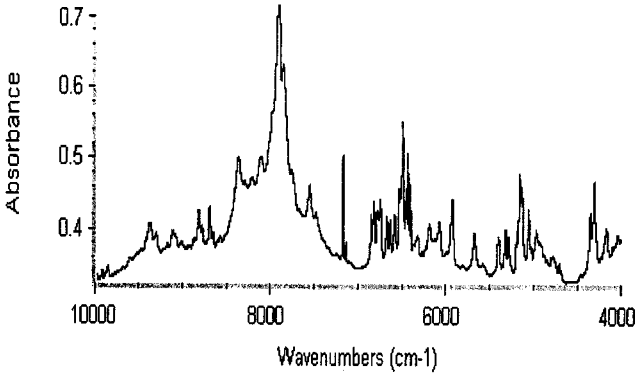

*KTA-1920x extended near-infrared diffuse reflectance secondary wavelength standard.*

### Novel approach to the theory of flow analysis

*Vladimir V. Kuznetsov, Yuliya V. Ermolenko and Mikhail L. Bykhovskii, Department of Analytical Chemistry, Mendeleyev University of Chemical Technology of Russia, 9 Miusskaja Square, 125047 Moscow, Russia*

A new approach to the theory of flow analysis is presented. This method is based on graph theory. The usage of ideas of graph theory to the description of flow systems allows a unit to link all of its parameters: chemical, configuration and instrumental features. The mathematical formulation of paths of passage from the initial node of the graph, allowing a substance to be determined, to the final one—the introduced product of analytical reaction—allows one deduce a key equation of the theory.

For the complexation reaction $M + R^* \rightarrow MR$, the equation is $h = 13(MR)c(R)D(MR) - 1 \sim (MR) \sim klc(M)$, where $h$ is a peak height, I $\sim$ (MR) the constant of stability, $c(R)$ the concentration of reagent, $D(MR)$ the dispersion coefficient, $\sim$(MR) the molar absorption coefficient, $\sim$ the extent of reaction, $k$ the instrumental factor, $l$ the thickness of the absorbing layer and $c(M)$ the concentration of metal to be determined.

The developed theory conforms to the main principles of chemical thermodynamics. This is fair because in each point of a flow there exists the local thermodynamic equilibrium. It allows one to find the optimal solutions at usage in flow systems of various analytical reactions. The examples of concrete usage of theory will be illustrated for redox reactions, ligand and heterogeneous exchange, where on-line concentration by co-precipitation is reviewed. It is also demonstrated that the conditions in a flow real-time system respond to the basic postulates of linear non-equilibrium thermodynamics.

The research was supported by grant from the Russian Foundation for Basic Research #00-03-32624.

### Development of portable near infrared (NIR) system using a microspectrometer

*Hyo-Jin Kim[1], Young-Ah Woo[1], Jae-Min Kim[2] and Myung-Yoon Kim[2], [1]College of Pharmacy, Dongduk Women's University, Seoul 136-714, [2]Spectron Tech. Co., Ltd, Seoul 136-132, Korea*

In recent years, a miniature spectrometer has been extensively developed due to the marriage of fiberoptics and semiconductor detector array. This type of miniature spectrometer has the advantages of low price and robustness due to the capability of mass production and no moving parts are required such as lenses, mirrors and a scanning monochromator. These systems are ideal for use in teaching laboratories, process monitoring and field analyses. A portable near infrared (NIR) system has been developed for qualitative and quantitative analysis. This system includes a tungsten halogen lamp as a light source, fiberoptics connected to the light source and a sample module to the microspectrometer. The size of spectrometer can be as small as $2.5 \times 1.5 \times 0.1$ cm. Wavelength ranges can be chosen as 360–800, 800–1100 and 1100–1900 nm depending on the type of detector. The software consists of various tools for multivariate analysis and pattern recognition techniques. To evaluate the system, long- and short-term stability, wavelength accuracy and stray light have been investigated and compared with a conventional scanning-type NIR spectrometer. This developed system can be sufficiently used for quantitative and qualitative analysis for various samples such as agricultural product, herbal medicine, food, petroleum, pharmaceuticals, etc.

### Versatile automatic GC system for large volume and conventional headspace analysis in pharmaceutical, forensic, food and beverage, and environmental control

*F. Pigozzo[1], G. Mapelli[1], F. Bedini[1], A. Trisciani[1], F. Munari[1], A. Sironi[1], R. Anderson[2] and D. Clay[2], [1]ThermoQuest Italia SpA, Strada Rivoltana, I-20090 Rodano, Milan, Italy, [2]ThermoQuest Corp., 2215 Grand Avenue Parkway, TX 78728, USA*

The great versatility of the headspace system allows this technology to be successfully applied in many application fields. In this perspective, two different approaches can be adopted as to the technique used to transfer the vapour sample from the sampling vial into the GC injector: static and dynamic. The advantages of the former towards the latter, besides the high reproducibility delivered, are the simple method development, ease of automation and a leak-free environment.

This paper describes how the high flexibility of a static headspace autosampler can be combined with different method requirements to cope superbly with the largest application range possible. In particular, applications in forensic, pharmaceutical, food and beverage and environmental control are described.

For these applications, a HS2000 automatic headspace autosampler was used. Its possibility to heat the syringe, heat and shake the sample in the incubation oven, and flush the barrel eliminates any problem related to sample condensation and cross-contamination due to carryover. Furthermore, the possibility to perform multiple subsequent injections with sample cryo-enrichment is another advantage, it being able to enhance sensitivity up to a factor of eight with respect to a conventional single-shot injection.

To test this technique, the HS2000 has been used in combination with two different cryofocusing systems: the Cold Trap 900 and the PTV injector. While the former is a separate piece of hardware that can be installed below either the SSL or the PTV injector, the latter is the PTV injector itself used as a cold sample inlet. Both cases showed good sensitivity, injected volume linearity and repeatability results. Moreover, it can house different sizes of vials and can perform the multiple headspace extraction technique. Real samples related to the determination of residual solvents in pharmaceutical bulks, blood alcohol analyses, pesticide screening and determination of wine aromas are presented and discussed.

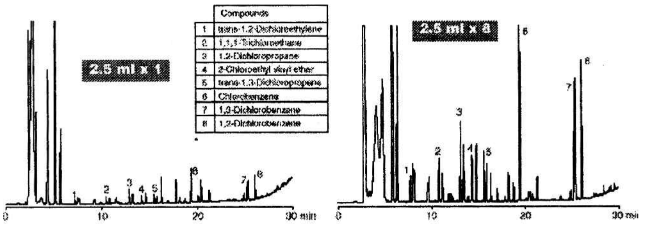

*Analysis of VOC in water according to EPA 601 using Cold Trap 900 at $-80\,^{\circ}C$ in combination with HS2000 and SSL injector. Gas chromatographic separation of 8 ml EPA 601 at 50 ppb: capillary column 30 m, 0.32 mm i.d., PS255 2 pm; HS2000 and split-splitless injection; column temperature programmed at $35\,^{\circ}C$ (6 min) to $150\,^{\circ}C$ at $6\,^{\circ}C\,min^{-1}$ to 250 at $20\,^{\circ}C\,min^{-1}$ (1 min); carrier gas, helium; detector, FID.*

### Flavour profiling of different olive oils with rancidity monitoring by thermal extraction GC/MS

*Andreas Hoffmann and Arnd Heiden, Gerstel GmbH & Co., KG, Aktienstra. 232–234, 45473 Melheim an der Ruhr, Germany*

Flavour is an important quality criterion for virgin olive oils. The identification of the compounds causing the flavour or off-flavour, therefore, is the key for quality control. Analysis in olive oils usually requires more or less cumbersome sample preparation like liquid–liquid extraction, solid-phase extraction or distillation techniques, often with the drawback of organic solvent use. Headspace and purge and trap methods do not use organic solvents, but their analyte range is restricted to the volatiles and, therefore, characterizes more the compounds contributing to the aroma/smell of a sample and not the flavour/taste.

Direct thermal extraction of olive oil in an inert atmosphere under controlled temperature conditions can be used to analyse easily the volatile and semivolatile compounds contributing to flavour. Olive oil (10 litres) is injected into an empty glass tube and inserted into a thermal desorption unit (TDS). The TDS was ramped to 80 °C with high desorption flow, allowing the analytes to be transferred into a PTV liner and trapped at sub-ambient temperatures (− 150 °C), while retaining the oil-matrix in the TDS tube. After completion of sample transfer, the PTV was ramped to 280 °C, releasing the extracted compounds into a GC-MS system for subsequent analysis at very high sensitivity.

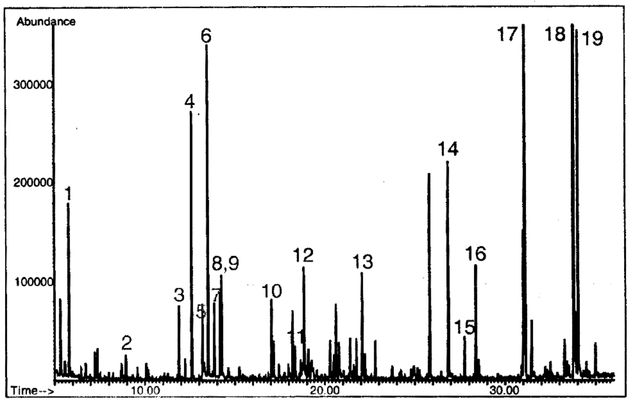

*Olive oil from Tuscany: 1, pentane; 2, pentanal; 3, hexanal; 4, octane; 5, hexyl methyl ether; 6, 2-hexenal; 7,* cis*-3-hexenol; 8,* trans*-2-hexenol; 9, hexanol; 10,* trans*-2-heptenal; 11, 2,4-heptadienal; 12,* cis*-3-hexenyl acetate; 13, nonanal; 14, 2-decenal; 15, 2,4-decadienal (isomer 1); 16, 2,4-decadienal (isomer 2); 17, copaene or z-cubebene; 18, z-farnesene; 19, z-muurolene or z-cadinene.*

### Fully automated preparative high-performance liquid chromatography using recycling technology

*Regis Boutant and Eric Verette, Gilson S. A., 19 avenue des Entrepreneurs, B. P. 45, F-95400 Villiers-le-Bel, France*

In the chemical industry, and particularly in the pharmaceutical industry, separation, purification and refining processes have a very important position and the quality requirements of the final products have drastically risen in the last decade. Among the techniques used in preparative high-performance liquid chromatography, recycling technology is definitely the best method to obtain the desired degree of purification performance in the separation of two closely eluted compounds such as isomers. Indeed, this technique presents quite interesting advantages about pressure and column length, particularly when using expensive chiral phases for the separation of enantiomers. In this work, a completely automated system is presented, which is composed of an injector, preparative pumps with new features, switching devices, a detector and a collector, all controlled by a centralized software. The system is applied to the separation of isomers to show the efficiency and robustness of the technique.

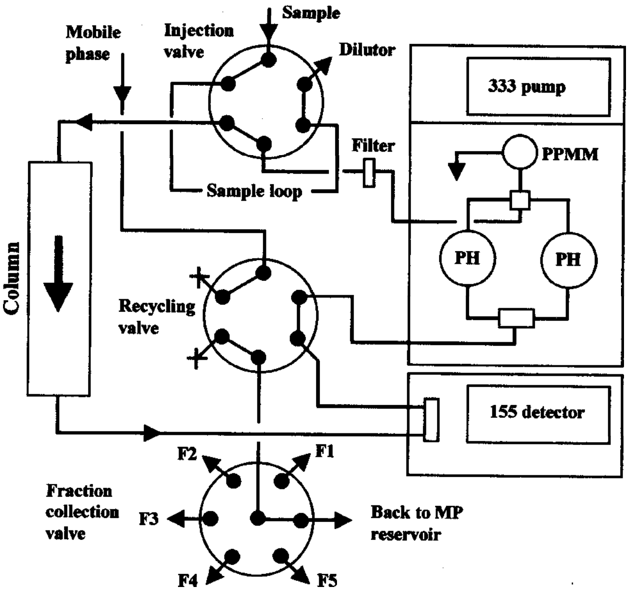

### HPLC analysis of teratrcycline and miconazolewa study in autosampler carry-over reduction

*Megan Thurmond and John C. Hodgin, Alcott Chromatography, Inc., 5680 Oakbrook Parkway, Suite 148, Norcross, GA 30093, USA*

Autosampler carry-over is the 'memory' of a previous sample injection found in a second, consecutive sample injection. It is usually expressed as a percentage of the compounds in one sample injection found in the consecutive injection of a blank solution. Carry-over causes both inaccuracy and irreproducibility in sample quantification. Two forms of carry-over can be described: physical and sorptive. Physical carry-over is due entirely to unswept places within the flow path of the injection system. A residual sample is trapped within the unswept places where it is slowly released with subsequent injections. Physical carry-over is usually eliminated simply by optimising the tubing connections and the plumbing network within the injection system to remove the unswept areas. Sorptive carry-over results from the adsorption of the sample within the injection system. Such adsorption has been detected within the walls of the sample loop [1, 2] and on the plastic rotor seals of the injection valve [3]. Sorptive carry-over can be minimised by judicious choice of solvents for both the mobile phase and sample solutions. These steps usually eliminate sorptive carry-over within the flow paths of the injection system, but do not necessarily remove sample residues from the injection ports or autosampler cannulas which can also contribute to carry-over. These areas often

1. Simonson, L., and Nelson, K., 1992, *LC/GC*, **10**, 533.
2. Fernadez-Otero, G. C., Lucangioli, S. E., and Carducci, C. N., 1991, *J. Chromatog.*, **654**, 87.
3. MacLeod, S. K., Fagan, D. T., and Scholl, J. P., 1991, *J. Chromatog.*, **502**, 22.

require additional rinsing steps for absolute carry-over removal.

The purpose of this presentation is to document the sorptive carry-over effects of the antibiotic tetracycline and the antifungal compound miconazol. Described in the presentation are the steps used for carry-over reduction and the design of a new autosampler, the Alcott Model 718ALD, which features an in-line injection port rinsing system used exclusively for carry-over reduction. This new in-line flushing system allows for a series of up to three rinse solvents to wash rapidly both the internal and external needle surfaces as well as the injection port of the sampling valve.

### Computer-assisted method validation and substantial time savings in carrying out the calculations and in compiling the validation report

*Wolf-Dieter Beinert[1], Reinhold Spatz[1] and Jean-Marc Roussel[2], [1]Merck KGaA, Germany, [2]Merck S.A., France*

All analytical methods applied in quality control laboratories must be validated. However, the calculation of the method criteria to be validated and the compilation of the validation report are often very tedious and time consuming.

Validation Manager is a validated and write-protected software dedicated to the validation of analytical methods. It can be used to calculate and assess the method criteria specificity, precision, accuracy, linearity, range, detection limit and quantitation limit. Moreover, it can be used to perform robustness studies or interlaboratory trials. The method criteria are calculated in accordance with the international guidelines of ICH, USP and ISO. Contents of the automatically compiled report are selectable, and both form and language of the report can be adapted by exporting it as a Word™ document. This validation report can be submitted directly to the authorities as a final validation document. In this way, Validation Manager helps substantially to save time in the method validation process.

The different steps of method validation with Validation Manager will be explained. Moreover, an estimation of the timesavings achieved by the use of a validation software will be presented.

### Automatic HPLC method development: faster method development and better methods using artificial intelligence and virtual chromatography

*Wolf-Dieter Beinert[1], Reinhold Spatz[1], Volker Eckert[1], Sergej Galushko[2] and Irina Shishkina[2], [1]Merck KGaA, Germany, [2]Institute of Bioorganic Chemistry and Petrochemistry, National Academy of Science of the Ukraine, Kiev, Ukraine*

An automated HPLC method development system has been developed for reversed-phase HPLC methods. It consists of an HPLC system and a software package for computer-assisted method optimization. The software was equipped with a control module for the HPLC system. In addition, a module of artificial intelligence has been added that is necessary to evaluate the experimental results and to take decisions during the method development process. Virtual chromatography (i.e. prediction of optimum HPLC conditions and simulation of chromatograms on the PC without to perform any prior chromatographic experiments) is used to reduce the necessary experiments to an absolute minimum. An automatic column and solvent switching function allows the optimization for each column/organic modifier combination and thus a quick statement to which column/solvent combination gives the best results. ChromSword Auto can thus realize substantial timesavings in the method development process and optimization methods with regard to minimum run time and maximum peak resolution. Several application examples show the potential of this innovative software technology for HPLC method-development laboratories.

### Automated sample preparation for creams, gels and ointments

*Lynn Jordan and Ed Yapchanyk, Zymark Corp., 68 Elm Street, Hopkinton, MA 01748, USA*

Sample preparation for creams, lotions, gels and ointments is a difficult task. Their high viscosity or unique rheology causes analyte extraction of these sample types to be particularly difficult to disperse. They are most troublesome when the semisolid base product is not miscible with the preferred solvent required for the assay. This poster will illustrate how automated sample preparation can be utilised to overcome these difficulties. One benefit of automated sample preparation is its ability to eliminate differences in various manual techniques such as sample dispersion. In addition, automated systems can provide a documented method and a complete audit trail of the sample handling which many laboratories require to meet 21 CFR Part 11 compliance. This poster will examine the parameters used for an efficient and reproducible sample preparation for these sample types, as well as the resulting documentation and audit trail.

### Fieldable highly-sensitive instrument for trace mercury detection in water

*Shiquan Tao, George P. Miller, Christopher Winstead, Chuji Wang and Fabio Mazzotti, Diagnostic Instrumentation and Analysis Laboratory, Starkville, MS 39759, USA*

Trace mercury in water samples presents a serious challenge to the analytical chemist. Mercury concentration in natural water (rain, river, ocean) is in the ppt level. In some contaminated water bodies, mercury concentration could be much higher. For environmental investigation and a contaminated site remediation test, a detection technique with a detection limit at the sub-ppb level is required. In the laboratory, ICP-MS or AAS combined with some preconcentration techniques can be used to analyse mercury in such a low concentration level. However, on-site mercury detection has many benefits for environmental research and contaminated site cleaning. In this report we present a highly sensitive fieldable instrument for the detection of ppt level mercury in water sample. The instrument consists of a mercury pen lamp as the light source, a laboratory build-up small-echelle spectrometer, a CCD detector and a laboratory build-up atomiser. Chemical reduction and membrane separation techniques are used in building up the atomiser. A detection limit of 1.2 ppt mercury in water is achieved by use the constructed system (see the figure).

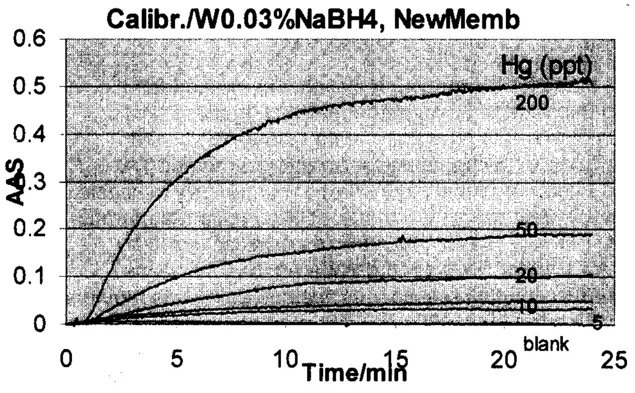

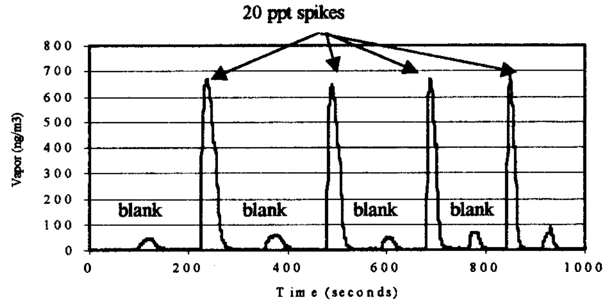

*Determination of ppt levels of mercury in water (blanks and 20 ppt spikes).*

**Determination of mercury in air, solid and liquid samples using GC/MS and a portable atomic absorption mercury analyser with Zeeman correction**

*Michael Markelov, Sergey Sholupov, Joseph Siperstein, Olga Bershevits and David Dechant, Ohio Lumex Co., 5405 E. Schaaf Road, Cleveland, OH 44131, USA*

The atomic absorption RA-915+ portable mercury analyser with Zeeman correction was interfaced with the ASIST-150-2N dual needle headspace autosampler. The reducing agent ($SnCl_2$) was added automatically to the headspace vial and equilibrated in the headspace oven. The vapor phase was swept into the spectrometer by nitrogen using a dual-needle arrangement or by pressurising the vial and letting the vapors expand into a multipath 10 m cell of the RA-915+. The data generated were quantitatively interpreted using several methods such as external standard, the method of standard additions and multiple headspace extraction.

The same headspace autosampler was also interfaced with a GC/MS system for analysis of mercury. It was shown that results obtained by GC/MS correlated well with those obtained with RA-915+. However, detection limits of the headspace GC/MS system were in the ppb range, while the sensitivities achieved with RA-915+ were in the ppt range.

This paper will concentrate on the conditions of headspace preparation and sampling for the generation of quantitative results. The methodologies of quantification in vapors and condensed matrices will be presented.

A headspace autosampler with atomic absorption gives an analyst a number of practical advantages including superior selectivity and sensitivity. This eliminates cross-contamination between samples, which is common problem with liquid autosamplers. Moreover, headspace sampling allows the analysis of solid samples without digestion in many cases.

**On-line flow system for lead enrichment and determination by flame atomic absorption spectroscopy**

*Sergio Luis Costa Ferreira*[1] *and Valfredo Azevedo Lemos*[1,2], [1]*Universidade Federal da Bahia, Instituto de Qufmica, Salvador-BA, 40170-290,* [2]*Universidade Estadual do Sudoeste da Bahia, Campus de Jequi6, Jequi6-BA, 45200-000 Brazil*

In the present paper, an on-line system for enrichment and determination of lead is proposed. It is based on the chemical sorption of lead(II) ions on a minicolumn packed with polyurethane foam loaded with 2-(2-benzothiazolylazo)-2-*p*-cresol (BTAC) reagent. After preconcentration, lead(II) ions are eluted by 0.10 mol l$^{-1}$ hydrochloric acid solution and determined directly by FAAS. Several parameters were studied. The results demonstrated that lead can be determinate with an enrichment factor of 26 for a sample volume of 7.0 ml and preconcentration time of 1 min. The detection limit (3 s) was 1.01 g$^{-1}$ and the precision (RSD) reached 6.0–0.7% in lead solutions of 10–500 l g$^{-1}$ concentration, respectively. The enrichment factor and the detection limit can be further improved by increasing preconcentration time. Achieved sampling rate was 48 samples h$^{-1}$.

The effect of other ions in concentrations agreeing with biological samples was studied. The results demonstrated that the proposed procedure has necessary selectivity for lead determination in seafoods and other biological samples. The accuracy of the procedure developed was confirmed by analysis of the following certified reference materials: fish tissue IAEA, lobster hepatopancreas NRCC TORT-1 and citrus leaves NIST 1572. Recoveries of spike additions (0.2 or 1.0 μg g$^{-1}$) to several seafood samples were quantitative (90–107%). These results proved also that the procedure is not affected by matrix interferences and can be applied satisfactorily for lead determination in polluted samples of shrimp, oyster, crab, fish and mussel.

**High-speed screening of dioxins in flue gases by using atmospheric pressure chemical ionisation ion-trap mass spectrometry**

*Yasuaki Takada, Yuichiro Hashimoto, Masuyoshi Yamada, Masao Suga and Minoru Sakairi, Central Research Laboratory, Hitachi Ltd, Kokubunji, Tokyo 185-8601, Japan*

Environmental pollution by dioxins (2,3,7,8-tetracloro-dibenzo-*p*-dioxin and its isomers) is a serious problem and threat to human health. The main emission sources of dioxins are incinerators. To reduce dioxin formation, highly controlled incinerator operation based on real-time monitoring of index compounds such as dioxin precursors is needed.

However, the correlation between the concentration of index compounds and the toxicity equivalent quantity (TEQ) of dioxins is not strong enough for accurate determination of TEQ in flue gases. However, high-resolution GC/MS analysis to determine TEQ takes >1 week due to its complicated pretreatment processes. Thus, it is difficult to determine the TEQ of an incinerator over a long period.

To monitor the TEQ of dioxins from incinerators, we developed a high-speed analysis system that enables the total amount of dioxins to be quickly determined. Because the correlation between the total amount of dioxins and TEQ is high, we can than accurately estimate TEQ. Our system consists of a pretreatment system with an absorbent, an atmospheric pressure chemical ionisation (APCI) ion source, and an ion-trap mass spectrometer (ITMS). Flue gases are introduced into the pretreatment system for several hours and chemicals in the gases are concentrated on the absorbent. These chemicals are then extracted and introduced into the highly sensitive APCI-ITMS. The analysis time after sampling is ~2 h, short enough to allow for frequent checking of the flue gases.

The work was supported by the New Energy and Industrial Technology Development Organisation.

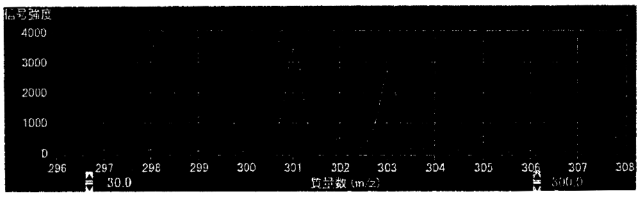

*Mass spectrum of 1,2,3,4,-tetraclorodibenzo-p-dioxin in room air. Concentration of the sample was ~3 μg Nm$^{-3}$ (M-CI + O) ions are clearly observed on the mass spectrum.*

### In-cylinder on-line measurement of exhaust gas compounds of combustion engines by mass spectrometry

*Marcus Gohl, Gerhard Matz and Wolfgang Schroeder, Technical University of Hamburg-Harburg, Environmental Measurement Technology, Harburger Schloss Str. 20, 21079 Hamburg, Germany*

An increasing environmental consciousness causes new regulations for emissions of exhaust gases. Although a reduction of many compounds of the exhaust gas of automobiles has already been achieved in recent years, new regulations will limit emissions to lower levels in the near future. For a better understanding of the process causing emissions, every phase of the combustion cycle has to be monitored on-line (A).

A new measurement system has been developed to monitor exhaust gas compounds in the exhaust pipe or directly in the cylinder. The on-line measurement system consists of the MS Kodiak 1200 (Bear Instruments) equipped with a high-temperature direct inlet. The MS contains an EI/CI source, a collision-cell and two mass analyser quadrupoles (A). The system has been extended by a digital signal processor for fast data acquisition. Operating in SIM mode, it is possible to monitor the concentration of various compounds of the exhaust gas with 12 500 samples s$^{-1}$ and resolve single four-stroke cycles.

To perform measurements, the spectrometer has been attached to the engine of a motor test stand. The inlet conducts the gas ($T_{gas}$ = 1000 K) directly from the combustion chamber with pressures of up to 20 bar via a skimmer to the ion source of the MS (B).

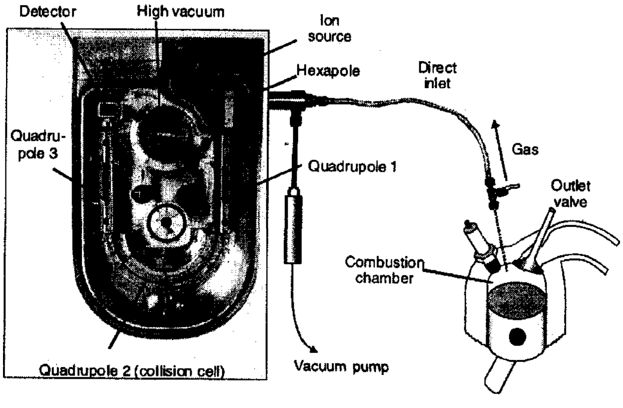

*Measurement system (A).*

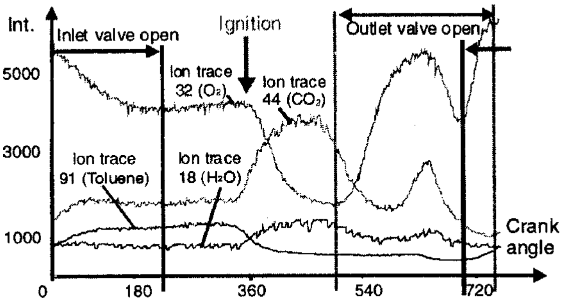

*In-cylinder measurement (B).*

### Automating the pressurised solvent extraction of oxygenated polyaromatic hydrocarbons from soils and river sediments

*Al Kazlunas[1] and Jiri Vejrosta[2], [1]Applied Separations, Inc., 930 Hamilton Street, Allentown, PA 18101, USA, [2]Institute of Analytical Chemistry, Academy of Sciences of the Czech Republic, Veveri 97, 611 42 Brno, Czech Republic*

The Soxhlet extraction of soil and sediment samples is a rugged laboratory technique using relatively inexpensive glassware, requiring no hands-on manipulation after loading, and providing efficient extraction. Unfortunately, Soxhlet extraction of polyaromatic hydrocarbons from soils may extend to 24 h and consume 300 ml of solvents.

This paper investigates the automation of the high-temperature extraction of oxygenated polyaromatic hydrocarbons (PAH) from soils and sediments as a fast alternative to the lengthy Soxhlet extraction. The optimised automation sequence of the extraction parameters and evaluation of serial and parallel sample processing schemes are discussed. In addition, an analysis of the time and cost savings inherent in the pressurised solvent extraction technique as a result of reduced solvent consumption and reduced manpower requirements is included.

Extraction efficiency of PSE versus Soxhlet oxygenated PAH:

- 9-Fluorenone: 104.4.
- 9,10-Anthracened ione: 106.8.
- 7H-benz(de)anthracen-7-one: 114.0.
- PSE/Soxhlet%.

### Automated stack gas monitoring system for total volatile organic halogen as a dioxin precursor

*Tsuneaki Maeda[1], Ikuo Watanabe[1], Sumio Goto[2] and Sukeo Onodera[3], [1]R&D Division DKK-TOA Corp., Kichijojikita-machi, Musashino-shi, Tokyo, [2]National Institute of Public Health, 4-6-1, Sirokanedai, Minato-ku, Tokyo, [3]Faculty of Pharmaceutical Sciences, Science University of Tokyo, 12 Ichigayafunagawara, Shinjyuku, Tokyo, Japan*

There exists a serious problem caused by emission of unexpected dioxin from stack gas. It is very difficult to monitor dioxin itself, but precursor monitoring is easier. Many precursors were listed and we investigated the relationship between dioxin reproductions after the filter. Many of the results showed a good relation, but there is no simple monitoring system for stack gas. We developed an automated monitoring system for total volatile organic halogen as a dioxin precursor.

In the first stage of the sampling, water is condensed and removed. Inorganic halogen is removed in this stage, then sample is introduced into the concentrator. Air and the remained inorganic gas replaced $H_2$, then it is thermally desorbed. All the collected organic compounds are introduced into the reduction furnace. It produces HCI and HBr. These are then solved in the solvent. The conductivity of the solvent changes with respect to the concentration of the halogen, which contained total volatile halogenated components (TVOX).

The figure shows the flow diagram of this system. Analysis cycle time is 15 min. The detection limit is sub-pg $m^{-3}$. The sample volume is adjusted to the concentration level of the incinerator. We also show the time deviation of TVOX emission from incinerator. CO and $O_2$ indicate the state of combustion, but most of all the dioxin produced after the combustion. TVOX represented the remained source of the dioxin. Thus, it is better indicator for the operation of the furnace.

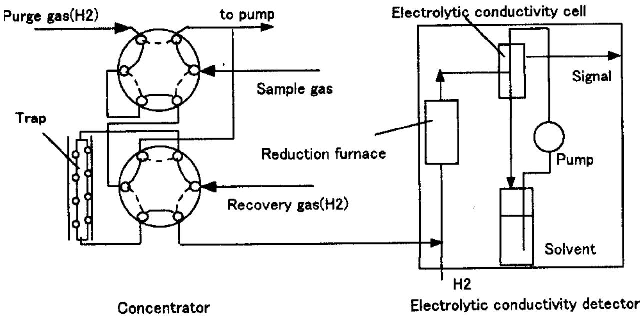

*Flow diagram of the TVOX monitoring system.*

### Optimisation of method parameters for the evaluation of USEPA Method 524.2 using a purge and trap with GC/MS

*Glynda A. Smith, Eric T. Heggs and Mark Krigbaum, Tekmar-Dohrmann, 7143 East Kemper Road, Cincinnati, OH 45249, USA*

Purge and trap coupled with gas chromatography offers a simple method for the extraction and concentration of volatile organic compounds (VOC) from multiple matrices such as water and soil. USEPA Method 524.2 monitors a wide range of VOC in drinking water.

Owing to the range and number of compounds analysed in Method 524.2, there are several analytical problems that can arise. One of the most common problems involves the transfer of water to the analytical column. Excessive water transfer can cause problems with both chromatography and the mass spectrometer.

In this paper, an evaluation of the different methods of moisture control will be presented and the Tekmar-Dohrmann 3100 Purge & Trap will be optimised for the analysis of the USEPA 524.2 compound list using GC/MS. The optimisation of method parameters will focus on achieving the optimal chromatography with the lowest detection limits and maximum reproducibility.

Presentation of results will include calibration curves, sample reproducibilities and minimum detection limits.

### Sequential automated analytical system for chlorophenols in drain water of flue gas from waste incinerators by using HPLC/ECD

*Ikuo Watanabe[1], Takashi Ikeguchi[1], Ryouko Nishimura[2], Chiaki Terashima[2], Kazuhiko Yamasaki[2] and Masahiro Furuno[2], [1]National Institute of Public Health, 4-6-1 Shirokane-dai Minato-ku, Tokyo 108-8638, [2]GL Sciences, Inc., 237-2 Sayamagahara, Irurna Saitama 358-0032, Japan*

Chlorophenols (CP) in flue gas from waste incinerators are noted as the precursors of polychlorinated dibenzo-*p*-dioxins (PCDD) and polychlorinated dibenzo-*p*-furans (PCDF). In addition, some of CP are relevant to environmental endocrine disruptors. Thus, the monitoring of CP in flue gas is very important and the sequential automated analytical system for CP containing two to three chlorine atoms was developed.

The system is composed of four parts, a cooled sampling unit for drain water, a periodical introduction unit for drain water sample to HPLC, an HPLC/ECD (electrochemical detector) for separation and detection of CP, and a control unit for each unit and data handling (see the figure). The sampling unit has two parallel traps of drain water for continual sampling and the sampling flow rate was adjusted to $2\,l\,min^{-1}$ with a mass flow controller.

The system was tested at a middle-sized incinerator for industrial waste (treatment capability $70\,ton\,day^{-1}$) for 10 days, and five CP were constantly detected at 10–1000 ng N $m^{-3}$. Then, the system was confirmed as being able to use the monitoring of CP in flue gas for a long period. However, we also found the slight effect of the previous sample adhering to the inner wall of a traps on the next sample determination and the relatively short life (1 week in the tested case) of the precolumn. These problems can be solved without much difficulty.

Columns 1 (50 mm) and 2 (150 mm); I, nertsill ODS-3, d.p. = 3 µm, i.d. = 2.1 mm sol.; methanol/water = 60/40, 5% $H_3PO_4$ det.; ECD, 1200 mV versus Ag/AgCl.

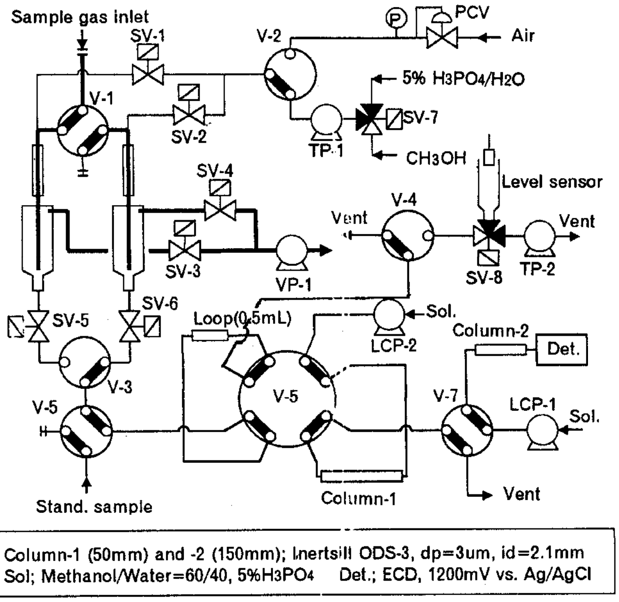

*Flow diagram of a sequential automated CPs analyser.*

